# A cancer-associated point mutation disables the steric gate of human PrimPol

**DOI:** 10.1038/s41598-018-37439-0

**Published:** 2019-02-04

**Authors:** Alberto Díaz-Talavera, Patricia A. Calvo, Daniel González-Acosta, Marcos Díaz, Guillermo Sastre-Moreno, Luis Blanco-Franco, Susana Guerra, Maria I. Martínez-Jiménez, Juan Méndez, Luis Blanco

**Affiliations:** 1 Centro de Biología Molecular “Severo Ochoa” (CSIC-UAM) c/Nicolás Cabrera 1, Cantoblanco, 28049 Madrid, Spain; 2 Centro Nacional de Investigaciones Oncológicas (CNIO), c/Melchor Fernández Almagro 3, 28029 Madrid, Spain

## Abstract

PrimPol is a human primase/polymerase specialized in re-starting stalled forks by repriming beyond lesions such as pyrimidine dimers, and replication-perturbing structures including G-quadruplexes and R-loops. Unlike most conventional primases, PrimPol proficiently discriminates against ribonucleotides (*NTPs*), being able to start synthesis using deoxynucleotides (dNTPs), yet the structural basis and physiological implications for this discrimination are not understood. *In silico* analyses based on the three-dimensional structure of human PrimPol and related enzymes enabled us to predict a single residue, Tyr^100^, as the main effector of sugar discrimination in human PrimPol and a change of Tyr^100^ to histidine to boost the efficiency of *NTP* incorporation. We show here that the Y100H mutation profoundly stimulates *NTP* incorporation by human PrimPol, with an efficiency similar to that for dNTP incorporation during both primase and polymerase reactions *in vitro*. As expected from the higher cellular concentration of *NTPs* relative to dNTPs, Y100H expression in mouse embryonic fibroblasts and U2OS osteosarcoma cells caused enhanced resistance to hydroxyurea, which decreases the dNTP pool levels in S-phase. Remarkably, the Y100H PrimPol mutation has been identified in cancer, suggesting that this mutation could be selected to promote survival at early stages of tumorigenesis, which is characterized by depleted dNTP pools.

## Introduction

Incorporation of ribonucleoside triphosphates (*NTPs*) onto DNA is generally considered harmful, because the persistence of ribonucleoside monophosphates (*NMPs*) in the DNA is associated with several potential problems: (1) *NMPs* are non-canonical templates for DNA replication^1–3^; (2) the assembly of nucleosomes is impeded by the presence of *NMPs*^4^; (3) *NMPs* embedded in the DNA are more prone to hydrolysis than deoxynucleosides monophosphates (dNMPs) and consequently render the DNA backbone more labile^5^; and (4) a single *NMP* embedded in the DNA duplex can result in helix perturbation and can alter protein recognition and binding^6,7^. Most DNA polymerases, in particular those specialized in bulk DNA replication, efficiently discriminate in favor of dNTPs, which reflects the hazardous potential of NTPs^2,8,9^. To distinguish *NTPs* from dNTPs, DNA polymerases are commonly endowed with “steric gates” formed by residues with bulky side chains, such as tyrosine or tryptophan, which sterically hinder the access of *NTP* into the active site via collision with the 2′ hydroxyl group (2′OH). However, the exclusive use of dNTPs by DNA polymerases is a difficult challenge because *NTPs* are far more abundant in cells than dNTPs^10^. Indeed, recent studies demonstrated that despite their ability to discriminate against *NTPs*, replicases incorporate these substrates at strikingly high rates *in vivo* (e.g. around 1 per 1 kb in the case of yeast Polε) because of their high cellular concentration^11^.

Nonetheless, this incorporation of *NTPs* is not necessarily hazardous as single embedded *NMPs* are efficiently removed by the ribonucleotide excision repair pathway^12^, which is initiated by RNase H2, an enzyme essential to preserve genomic stability in mammals^13^. Interestingly, likely due to the transient nature of *NMPs* in DNA, the incorporation of *NTPs* into DNA is physiologically relevant and even beneficial in several biological processes, for example by contributing to mismatch repair signalling^14,15^, improving the fidelity of Polμ-mediated non-homologous end joining *in vitro*^16^, mediating error-free tolerance of 8-oxo-dexyguanosine (8oxoG) in *Schizosaccharomyces pombe*^17^, and even mediating mating-type switching also in fission yeast^18^. Overall, specialized polymerases involved in translesion synthesis (TLS) can exploit a relaxed steric gate, allowing the use of ribonucleotides as alternative substrates to bypass DNA lesions^19^. A special scenario implies the recurrent synthesis of short chains of *NMPs* (RNA primers) generated by conventional primases to prime DNA replication that are accurately removed^20–22^.

PrimPol is a novel human primase/polymerase belonging to the Archeal-Eukaryotic-Primase (AEP) superfamily^23^ that is specialized in re-priming at stalled forks to re-start DNA replication downstream of UV damaged sites^24,25^, G quadruplexes^26^ and even R-loops^27^. PrimPol, which localizes to both mitochondria and nuclei of human cells, displays both primase and polymerase activities^23^. As a polymerase, PrimPol efficiently tolerates different DNA template lesions *in vitro* by either incorporating nucleotides opposite them or beyond the damaged site in the case of unreadable lesions such as pyrimidine dimers^23,24^; however, the physiological relevance of this polymerase activity is not well understood. Conversely, it is well established that PrimPol primase activity is relevant to mediate replication re-start at stalled forks^24,28^, and this appears to be its main role *in vivo*. Whereas PrimPol accepts both ribo and deoxynucleotides at the initiation site^29^, unlike unconventional primases it efficiently discriminates against *NTPs* at the elongation site, to incorporate dNTPs with much higher efficiency^23^. Accordingly, human PrimPol must have structural elements to discriminate against the use of *NTPs*, although the basis for such discrimination and the physiological relevance of PrimPol preference for dNTP incorporation remain unknown.

In this work, we have identified a single residue, Tyr^100^, as the main mediator of sugar discrimination in human PrimPol. Structural analyses and sequence comparison with other members of the AEP superfamily enabled us to predict and demonstrate that a change of Tyr^100^ to histidine frees PrimPol to efficiently incorporate *NTPs* during polymerase and primase reactions *in vitro*, and provides increased resistance to downregulation of dNTP pools during DNA replication in murine embryonic fibroblasts (MEFs) and U2OS osteosarcoma cells. Remarkably, the very same mutation in PrimPol (Y100H) has been identified in lung cancer^30^, suggesting its implication to promote survival during the tumorigenesis process.

## Results

### Identification of Tyr^100^ as a potential sugar selector in human PrimPol

**Figure 1:**
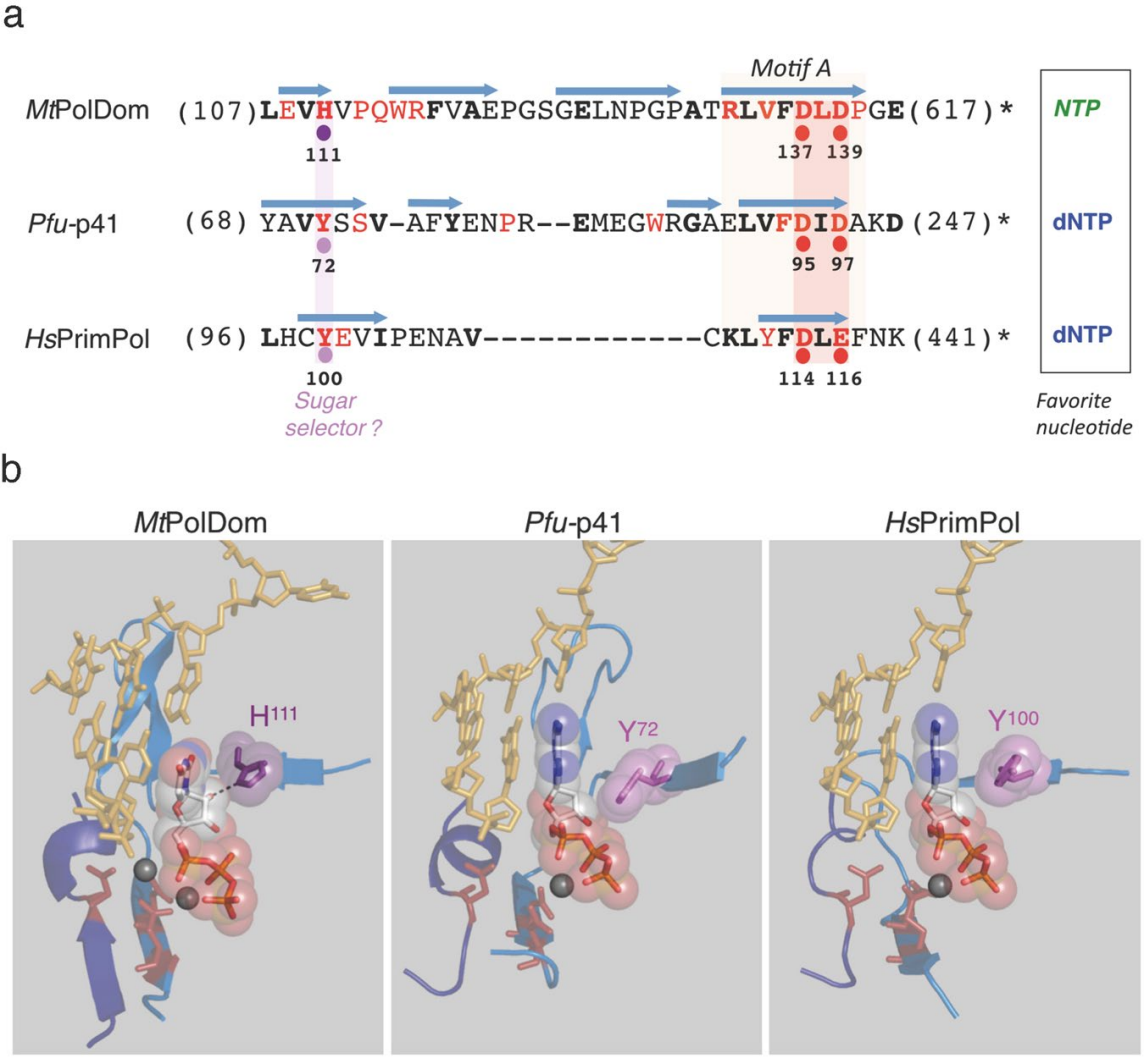
Prediction of the sugar selector in human PrimPol. (**a**) Primary sequence comparison of the region encompassing motif A of *Mt*PolDom, *Pfu-*p41 and *Hs*PrimPol. Catalytic residues involved in metal binding are indicated with red dots. Single residues acting as a sugar selector favoring *NTPs* or dNTPs are indicated with violet or pink dots, respectively; β-strands are indicated as light blue arrows. Figures in parenthesis indicate the number of N-terminal or C-terminal amino acid residues that are not shown. Invariant (red) or conserved (bold black) residues are indicated (see also Supplemental Fig. 1). (**b**) Structural details of the region aligned in part a, containing candidate residues to act as sugar selectors, and two catalytic metal ligands; a third metal ligand, embedded in an additional peptide segment (motif C; depicted in dark blue) is also shown in *Mt*PolDom (PDB ID: 3PKY, DNA template/primer from 4MKY and *NTP* from 3PKY), *Pfu*-p41 (PDB ID: 1G71, DNA template/primer and dNTP from 5L2X) and *Hs*PrimPol (PDB ID: 5L2X, DNA template/primer and dNTP from 5L2X).

To identify the residue/s that mediate sugar discrimination in human PrimPol we compared the primary sequences of biochemically characterized and crystallized AEP members with preference for either dNTP or *NTP* incorporation. *Pyrococcus furiosus* p41 (*Pfu-*p41), an archeal primase/polymerase^31–33^, was chosen as an AEP with preference for dNTPs, similar to human PrimPol^34^; in turn, *Mycobacterium tuberculosis* PolDom (*Mt*PolDom), the polymerase domain in Ligase D, a multi-domain protein specialized in non-homologous end joining (NHEJ)^35–37^, was chosen as an AEP with preference for *NTP* insertion. Multiple alignment of the primary sequence encompassing the highly conserved Motif A and its upstream flanking region (Fig. 1a) showed that *Mt*PolDom His^111^, a candidate residue to facilitate *NTP* incorporation^35–37^, is not conserved but substituted by a tyrosine in *Pfu-*p41 (Tyr^72^) and human PrimPol (Tyr^100^).

Analysis of the crystal structure of *Mt*PolDom indeed demonstrated that His^111^ interacts with the 2′OH group of the sugar moiety of the incoming *NTP*, possibly mediating its stabilization as a preferred substrate (Fig. 1b). Remarkably, *Pfu-*p41 Tyr^72^ and human PrimPol Tyr^100^ are located in a similar position to *Mt*PolDom His^111^ in their respective crystal structures (Fig. 1b), suggesting that they could also affect sugar selection. Moreover, the fact that tyrosines are bulkier than histidines supports a role in excluding *NTPs* by steric hindrance rather than stabilizing these substrates in the active site, which could explain the difference in sugar selectivity between human PrimPol/*Pfu-*p41 and *Mt*PolDom. Notably, *Mt*PolDom His^111^, *Pfu-*p41 Tyr^72^ and human PrimPol Tyr^100^, are all highly conserved among orthologs through evolution (Supplemental Fig. 1), further supporting a relevant role for these residues in substrate selection. Taken together, these *in silico* analyses suggest that human PrimPol Tyr^100^ could be a relevant mediator of sugar discrimination, and that mutation of this residue to histidine could boost *NTP* incorporation. Remarkably, a previous study^30^ compiled in the COSMIC database (http://cancer.sanger.ac.uk/cosmic)^38^, identified the Y100H mutation of human PrimPol in a lung tumor sample, which further prompted us to evaluate the effect of this mutation on sugar discrimination.

### Mutant Y100H can efficiently elongate primers with *NTPs*

**Figure 2:**
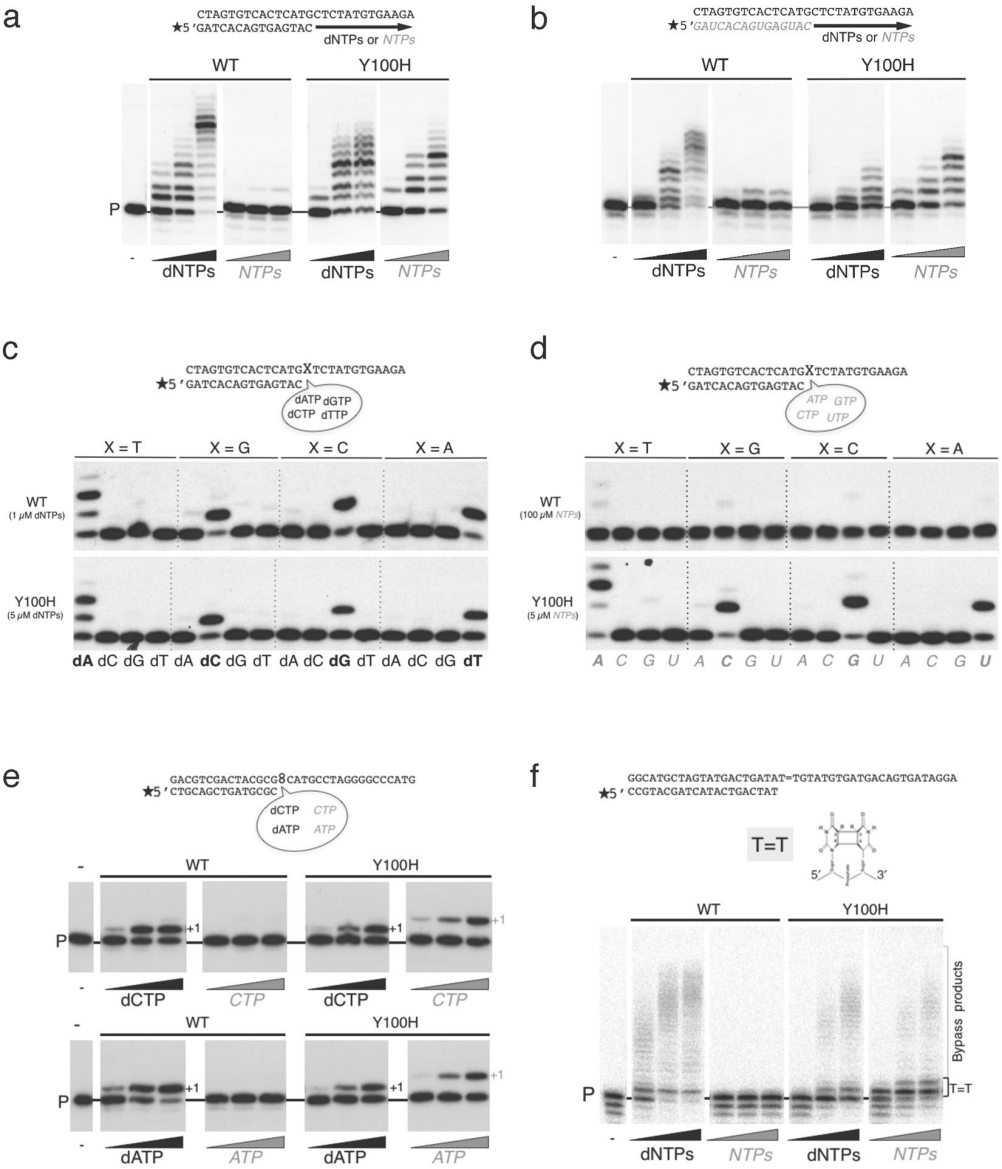
Use of ribonucleotides by the Y100H variant during primer extension and translesion synthesis. (**a**) DNA primer extension assay on the indicated template/primer by either wild-type (WT) PrimPol or Y100H, using increasing concentration of dNTPs (1, 10, 50 µM) or *NTPs* (1, 10, 50 µM). (**b**) RNA primer extension on the indicated template/ RNA primer structure as described in part a. (**c**) Matched versus mismatched nucleotide insertion at the four template bases (indicated as X in the scheme). Nucleotide insertion on each template-primer was analyzed in the presence of each individual dNTP at 1 µM or 5 µM for the WT or Y100H mutant respectively. (**d**) As described in (**c**), using NTP as substrates at 100 µM or 5 µM for the WT or Y100H mutant respectively. (**e**) Lesion bypass of 8oxoG (scheme of the template/primer structure at the top) by either WT PrimPol or Y100H (100 nM) in the presence of 100 µM MnCl_2_ as metal cofactor with increasing concentrations (1, 10, 50 µM) of dCTP or *CTP* (upper panel) and dATP or *ATP* (lower panel). (**f**) Lesion bypass of a CPD lesion (scheme of the template/primer structure at the top) of either WT PrimPol or Y100H with increasing concentrations of dNTPs or *NTPs* (1, 10, 100 µM). Full length gels corresponding to parts a, b, e and f are shown in Supplemental Fig. 5. The autoradiographs shown in this figure are representative of at least 3 independent experiments.

The effect of the Y100H mutation on sugar discrimination was firstly tested in primer extension *in vitro* assays, in which either the purified mutant or the wild-type (WT) PrimPol were incubated with a labelled DNA primer/DNA template molecule and either dNTPs of *NTPs* as substrates. As previously reported^23,39^, WT PrimPol could efficiently extend the primer using dNTPs, and displayed barely detectable activity using *NTPs* (Fig. 2a). In striking contrast, the Y100H mutation markedly increased the efficiency of *NTP* incorporation, which reached levels similar to those for dNTP insertion (Fig. 2a), demonstrating that the mutation enhances *NTP* incorporation in DNA primer extension reactions. Of note, the Y100H mutation slightly hampered dNTP incorporation when compared with the WT control (Fig. 2a), further suggesting that Tyr^100^ is relevant for the optimal incorporation of dNTP substrates.

We have previously showed that PrimPol can also extend RNA primers paired to DNA templates *in vitro*, suggesting a potential uncharacterized function in assisting transcription^39^. Thus, we sought to explore the effect of the Y100H mutation in this transcription-like reaction. As expected when the DNA primer was replaced by an equivalent RNA primer (Fig. 2b), WT PrimPol preferably extended the RNA primer with dNTPs. Interestingly, the Y100H mutation largely stimulated the efficiency of RNA primer extension with *NTPs* (Fig. 2b), overpassing the efficiency of RNA primer extension with dNTPs, thereby demonstrating the relevance of Tyr^100^ to regulate sugar selection by PrimPol.

Overall, the Y100H mutation did not significantly affect the proper base selection of both incoming dNTPs (Fig. 2c) and NTPs (Fig. 2d), preferentially inserting the complementary (Watson-Crick) nucleotide dictated by the first available template base. Nevertheless, under the assay conditions used, Y100H made less errors than WT PrimPol when using relatively similar amounts of dNTPs (Fig. 2c), but more errors with NTPs, even when WT PrimPol was assayed at a 20-fold higher NTP concentration (Fig. 2d). As it will be shown later in this section, this alteration in the sugar selectivity of inserted errors is the direct consequence of a more equilibrated NTP/dNTP discrimination ratio than that displayed by WT PrimPol. Accordingly, the level of ribonucleotide errors inserted *in vivo* by the Y100H variant is expected to be even higher, given the superior concentration of ribonucleotides versus deoxynucleotides inside cells. *In vitro* analysis of misinsertion by Y100H using physiological concentrations of ribonucleotides^10^ supports this prediction (see Supplemental Fig. 2).

Although the physiological relevance of PrimPol polymerase activity is not well understood, it is thought to be associated with a role in damage tolerance, by virtue of PrimPol efficient TLS activity *in vitro*^23,40^. A relevant scenario is the bypass of 8-oxoguanine (8oxoG), a highly pre-mutagenic lesion that PrimPol tolerates by incorporating dCTP (error-free) or dATP (error-prone) with similar efficiency to the insertion of dCTP opposite an undamaged dG^23,41^; Fig. 2e). Interestingly, and due to its improvement in *NTP* incorporation, the Y100H mutant was not only able to insert dCTP and dATP opposite the lesion, but also the corresponding ribonucleotides CTP and ATP (Fig. 2e).

To unravel the basis for sugar discrimination by PrimPol, and also for the improvement in *NTP* incorporation produced by the mutation Y100H, we performed steady-state kinetic analyses of deoxy *versus* ribonucleotide incorporation opposite either undamaged template bases dG and dT or 8oxoG. These studies demonstrated that WT PrimPol discriminates against ribonucleotides (*NTP*/dNTP discrimination values were in the range of 0.05 to 0.01; see Table 1) mainly due to their lower affinity (higher K_m_) compared with dNTPs: 11-fold (*CTP*/dCTP opposite dG), 47-fold (*ATP*/dATP opposite dT), 41-fold (*CTP*/dCTP opposite 8oxoG), and 28-fold (*ATP*/dATP opposite 8oxoG). As the k_cat_ values for *NTP versus* dNTP incorporation were only moderately decreased (2.3- to 6-fold), we conclude that a steric gate is the main responsible for the human PrimPol discrimination against ribonucleotides. A similar conclusion can be inferred from a previous report studying sugar selectivity of human PrimPol^42^.

**Table 1:** Pre-steady state kinetic parameters for deoxy- vs ribonucleotide incorporation.

Template	PrimPol	NTP or *dNTP*	K_m_ (µM)	k_cat_ (s^−1^)	Catalytic efficiency k_cat_/K_m_ (s^−1^ µM^−1^)	Discrimination^a^ (*NTP vs* dNTP)	Improved^b^ *NTP* preference	Improved^c^ *NTP* Binding
dG	WT	*CTP*	9.08 ± 0.87	(3.08 ± 0.04) · 10^−3^	(0.34 ± 0.04) · 10^−3^	0.05	**32-fold**	**13-fold**
		dCTP	0.84 ± 0.05	(5.83 ± 0,11) · 10^−3^	(6.94 ± 0.57) · 10^−3^			
	Y100H	*CTP*	**0**.**69 ± 0**.**08**	(3.24 ± 0.11) · 10^−3^	(**4**.**73 ± 0**.**73**) · **10**^**−3**^	**1**.**58**		
		dCTP	0.92 ± 0.20	(2.75 ± 0.17) · 10^−3^	(3.00 ± 0.83) · 10^−3^			
dT	WT	*ATP*	38.68 ± 3.64	(0.97 ± 0.02) · 10^−3^	(0.03 ± 0.003) · 10^−3^	0.01	**72-fold**	**36-fold**
		dATP	0.78 ± 0.12	(3.18 ± 0.13) · 10^−3^	(4.05 ± 0.80) · 10^−3^			
	Y100H	*ATP*	**1**.**06 ± 0**.**12**	(2.67 ± 0.09) · 10^−3^	(**2**.**51 ± 0**.**36**) · **10**^**−3**^	**0**.**72**		
		dATP	0.65 ± 0.21	(2.26 ± 0.18) · 10^−3^	(3.47 ± 1.42) · 10^−3^			
8oxodG	WT	*CTP*	34.36 ± 2.70	(1.72 ± 0.04) · 10^−3^	(0.05 ± 0.005) · 10^−3^	0.01	**173-fold**	**29-fold**
		dCTP	0.83 ± 0.13	(3.91 ± 0.19) · 10^−3^	(4.72 ± 0.98) · 10^−3^			
	Y100H	*CTP*	**1**.**17 ± 0**.**10**	(2.36 ± 0.07) · 10^−3^	(**2**.**01 ± 0**.**23**) · **10**^**−3**^	**1**.**73**		
		dCTP	2.56 ± 0.65	(2.98 ± 0.35) · 10^−3^	(1.16 ± 0.43) · 10^−3^			
	WT	*ATP*	20.86 ± 5.68	(0.62 ± 0.03) · 10^−3^	(0.03 ± 0.01) · 10^−3^	0.01	**75-fold**	**21-fold**
		dATP	0.75 ± 0.09	(3.72 ± 0.12) · 10^−3^	(4.95 ± 0.73) · 10^−3^			
	Y100H	*ATP*	**1**.**01 ± 0**.**22**	(2.49 ± 0.17) · 10^−3^	(**2**.**47 ± 0**.**72**) · **10**^**−3**^	**0**.**75**		
		dATP	0.75 ± 0.19	(2.45 ± 0.17) · 10^−3^	(3.27 ± 1.06) · 10^−3^			

WT PrimPol or Y100H mutant kinetic parameters (Km, kcat, and catalytic efficiency) were calculated for the insertion of single nucleotides (CTP, dCTP, ATP and dATP) opposite a templating G, T or 8oxoG, as described in Methods. ^a^NTP/dNTP discrimination ratio is calculated by dividing the catalytic efficiency of ribo vs deoxy versions of a given nucleotide. ^b^Improved NTP preference ratio (Y100H mutant relative to WT PrimPol) is calculated by dividing the discrimination values for a given nucleotide. ^c^Improved NTP binding ratio (Y100H mutant relative to WT PrimPol) is calculated by dividing the Km values for a given ribonucleotide.

In contrast with WT PrimPol, mutant Y100H displayed a more equilibrated *NTP*/dNTP discrimination ratios (between 0.72 and 1.73) resulting in a 32-fold to 173-fold improvement in *NTP* insertion (see Table 1). This was mainly due to a gain in affinity for *NTPs* (a large decrease in K_m_) relative to WT PrimPol: 13-fold (*CTP* opposite dG), 36-fold (*ATP* opposite dT), 29-fold (*CTP* opposite 8oxoG), and 21-fold (*ATP* opposite 8oxoG). The k_cat_ values for *NTP* incorporation (Y100H *vs* WT PrimPol) were only slightly improved (1- to 4-fold). These data support the role of Tyr^100^ as a steric gate residue, which defines PrimPol’s preference for dNTPs as incoming nucleotides. Interestingly, the catalytic efficiency for dNTP incorporation was only moderately decreased as a consequence of the Y100H mutation, with the exception of the insertion of dCTP opposite 8oxoG (4.1-fold), which is largely due to a 3-fold increase in K_m_. This suggests that Tyr^100^ is specifically required to optimize the error-free insertion of dCTP opposite 8oxoG.

PrimPol efficiently bypasses other lesions such as apurinic/apyrimidinic sites^23^ and thymidine dimers such as cyclobutane pyrimidine dimers (CPD) or 6–4 photoproducts^24^. Unlike TLS on 8oxoG, the bypass of these other lesions relies on PrimPol’s exceptional capacity to realign a matched primer terminus at downstream microhomologies found beyond the lesion, a process referred to as pseudo-TLS^24,39^. Remarkably, WT PrimPol was unable to catalyze pseudo-TLS with NTPs for bypassing CPDs (Fig. 2f), abasic sites or a 6–4 photoproducts (Supplemental Fig. 3). Conversely, the improvement in *NTP* binding by the Y100H mutation facilitated the efficient bypass of these lesions with ribonucleotides (Fig. 2f and Supplemental Fig. 3). Of note, the mutation again decreased the overall efficiency of TLS with dNTPs as compared with the WT control (Fig. 2f and Supplemental Fig. 3).

In conclusion, these analyses demonstrate that the Y100H mutation greatly increases the efficiency of *NTP* incorporation during primer extension *in vitro* by PrimPol, facilitating the use of these substrates also during PrimPol-mediated TLS.

### The Y100H mutation allows the efficient synthesis of RNA primers

**Figure 3:**
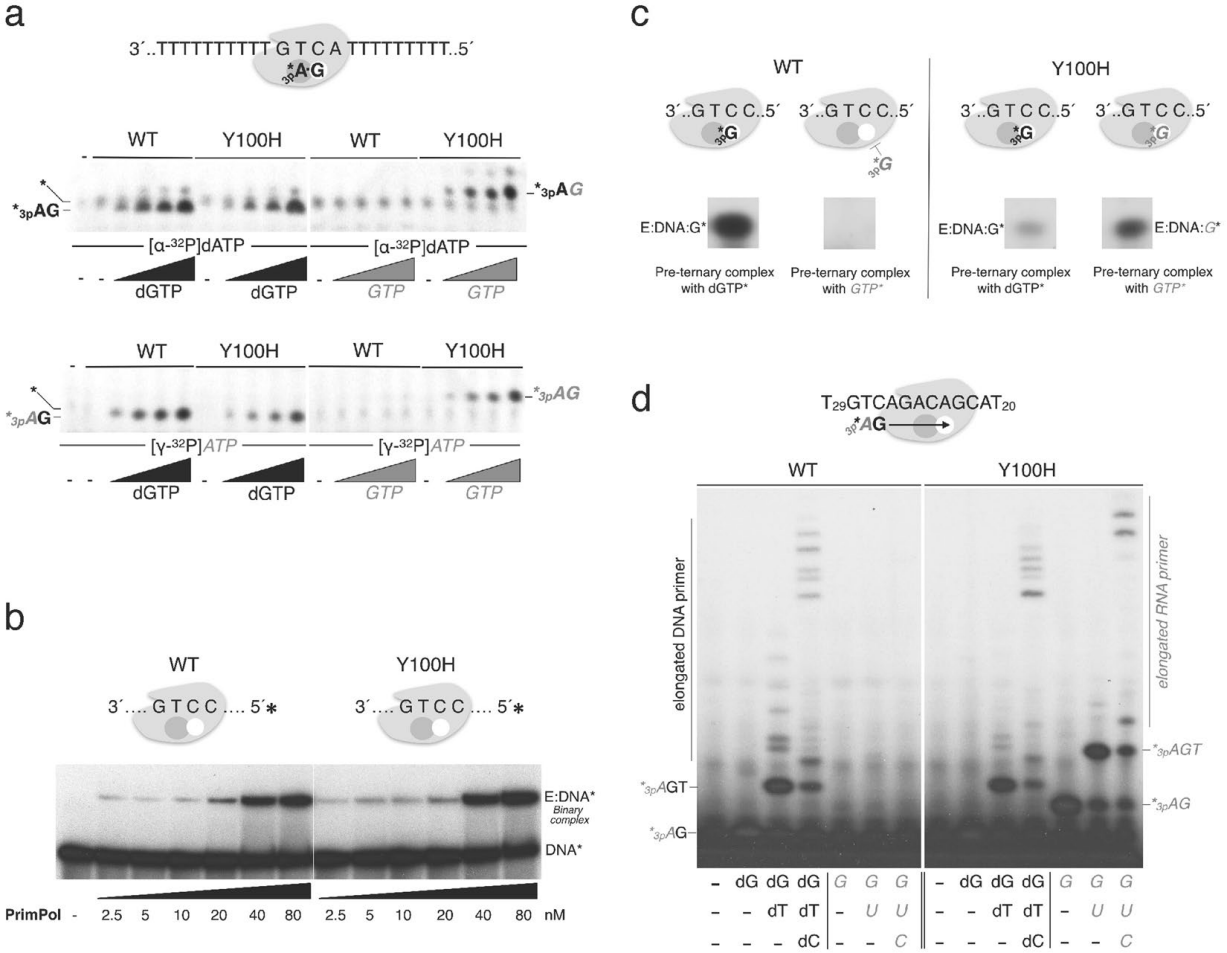
Ribonucleotides are valid substrates for the Y100H variant during primer synthesis. (**a**) Scheme on the top shows PrimPol in complex with the GTCA template oligonucleotide and the two nucleotides forming the initial dimer. The autoradiograph shows dimer formation (primase activity) either by wild-type (WT) PrimPol or Y100H (400 nM) using [α-^32^P]dATP (upper panel) or [γ-^32^P]*ATP* (lower panel) as the 5′-site nucleotide (16 nM), and increasing concentrations of either dGTP or *GTP* as the incoming 3′-site nucleotide (0, 10, 50, 100 µM). (**b**) Binary complex formation, measured by EMSA, between WT PrimPol or Y100H and labeled 60-mer DNA template GTCC (1 nM), using the indicated PrimPol concentration (2.5, 5, 10, 20, 40 and 80 nM) (**c**) Pre-ternary complex formation measured by EMSA between WT PrimPol or Y100H (1 µM), 60-mer DNA template GTCC and either [α-^32^P]dGTP or [α-^32^P]*GTP* (16 nM). (**d**) DNA or RNA primers synthesized using as template 5′-T_20_ACGACAGACTGT_29_ -3′ to allow elongation beyond the dimer. Products were labeled with [γ-^32^P]*ATP* (16 nM) as the 5′nucleotide and each subsequent nucleotide (dGTP, dTTP, dCTP) was added (10 µM) as indicated in the figure. Full length gels corresponding to parts a to d are shown in Supplemental Fig. 6. The autoradiographs shown in this figure are representative of at least 3 independent experiments.

To evaluate the effect of the human PrimPol Y100H mutation on sugar discrimination during initiation of primer synthesis, we used a 29-mer ssDNA template containing a single and preferred priming site (3′-GTCA-5′), flanked by homopolymeric tracks of Ts. PrimPol efficiently starts synthesis on this template by generating a *3pA*dG dinucleotide opposite the TC templating bases (see scheme in Fig. 3a), which is the initial and rate-limiting catalytic step during primer synthesis^29^. To form the initiating dimer, both WT PrimPol and mutant Y100H similarly used [α-^32^P]dATP at the 5′ initiation site and dGTP at the 3′ elongation site, but only the Y100H mutant formed a dimer when dGTP was replaced by *GTP* (Fig. 3a, upper panel). Thus, in agreement with the conclusions drawn from the primer extension assays, the Y100H mutant lacks ribose discrimination at the 3′ site also during dimer formation. As expected from the lack of sugar discrimination at the initiation site, both WT PrimPol and Y100H could efficiently catalyze the generation of a *3pA*dG dinucleotide (using [ϒ-^32^P]*ATP* instead of [α-^32^P]dATP at the 5′ position) and dGTP at the 3′ position (Fig. 3a, lower panel); but again, only mutant Y100H was able to use *GTP* at the 3′ site, generating the dimer *3pAG*. Altogether, these results indicate that sugar selection by WT PrimPol during dimer formation requires Tyr^100^, but it only operates on the elongating nucleotide (3′site). Replacement of Tyr^100^ by a histidine abolishes this discrimination and balances the efficiency of dNTP and *NTP* insertion.

To delineate the basis for sugar discrimination during primer synthesis by WT PrimPol or the Y100H variant, we next analyzed two individual steps preceding dimer formation^29^. Analysis of enzyme:ssDNA binary complex formation, as determined by EMSA with a ssDNA template containing a preferred and single priming site (3′-GTCC- 5′; see Methods), demonstrated that WT PrimPol and mutant Y100H have the same affinity for ssDNA (Fig. 3b). After forming a stable binary complex, and before catalyzing the initiating dimer, most primases firstly bind the 3′-site nucleotide (at the elongation site) and subsequently the 5′-site nucleotide (at the primer site). Formation of such a transient pre-ternary complex with the 3′ nucleotide has been recently demonstrated for human PrimPol using EMSA assays with radiolabeled nucleotides^29^. Thus, we could analyze sugar discrimination during pre-ternary complex formation by performing EMSA with a 60-mer ssDNA template [3′-GTCC-5′] and labelled [α-^32^P]dGTP or [α-^32^P]*GTP*. As shown in Fig. 3c (left panel), WT PrimPol was only able to produce a pre-ternary complex with [α-^32^P]dGTP, indicating that a steric gate precludes *GTP* binding at the 3′ site. Conversely, the Y100H mutant could form a stable pre-ternary complex using a ribonucleotide ([α-^32^P]*GTP*; see Fig. 3c, right panel), hence explaining the enhanced primase activity of the mutant using ribonucleotides. However, the Y100H mutation decreased formation of a pre-ternary complex with [α-^32^P]dGTP (as compared with WT PrimPol), suggesting that Tyr^100^ is required for maximal stability of dNTPs at the elongation site also during primer synthesis.

Finally, to analyze the formation of mature primers, we used a similar primase assay but this time with a variation of the DNA template sequence (3′-GTCAGACAGCA-5′) flanked by polydT tails (Fig. 3d). Consistent with the previous result, WT PrimPol and Y100H were able to make similar amounts of dimers, trimers and further elongated DNA primers when provided with the necessary deoxynucleotides (Fig. 3d). By contrast, only mutant Y100H was able to initiate and elongate RNA primers, reaching a similar and even longer mature size as DNA primers on the template sequence (Fig. 3d).

As shown here, and unlike WT PrimPol, the Y100H variant is able to make a full primer with *NTPs*, as a result of its loss of sugar discrimination at the 3′site together with PrimPol´s ability to extend an RNA primer. Thus, considering the high *in vivo* concentration of *NTPs*, it is tempting to speculate that the tumoral Y100H variant would execute its re-priming function during replication stress by synthesizing all-RNA primers.

### Y100H variant is competent for re-priming *in vivo*

**Figure 4:**
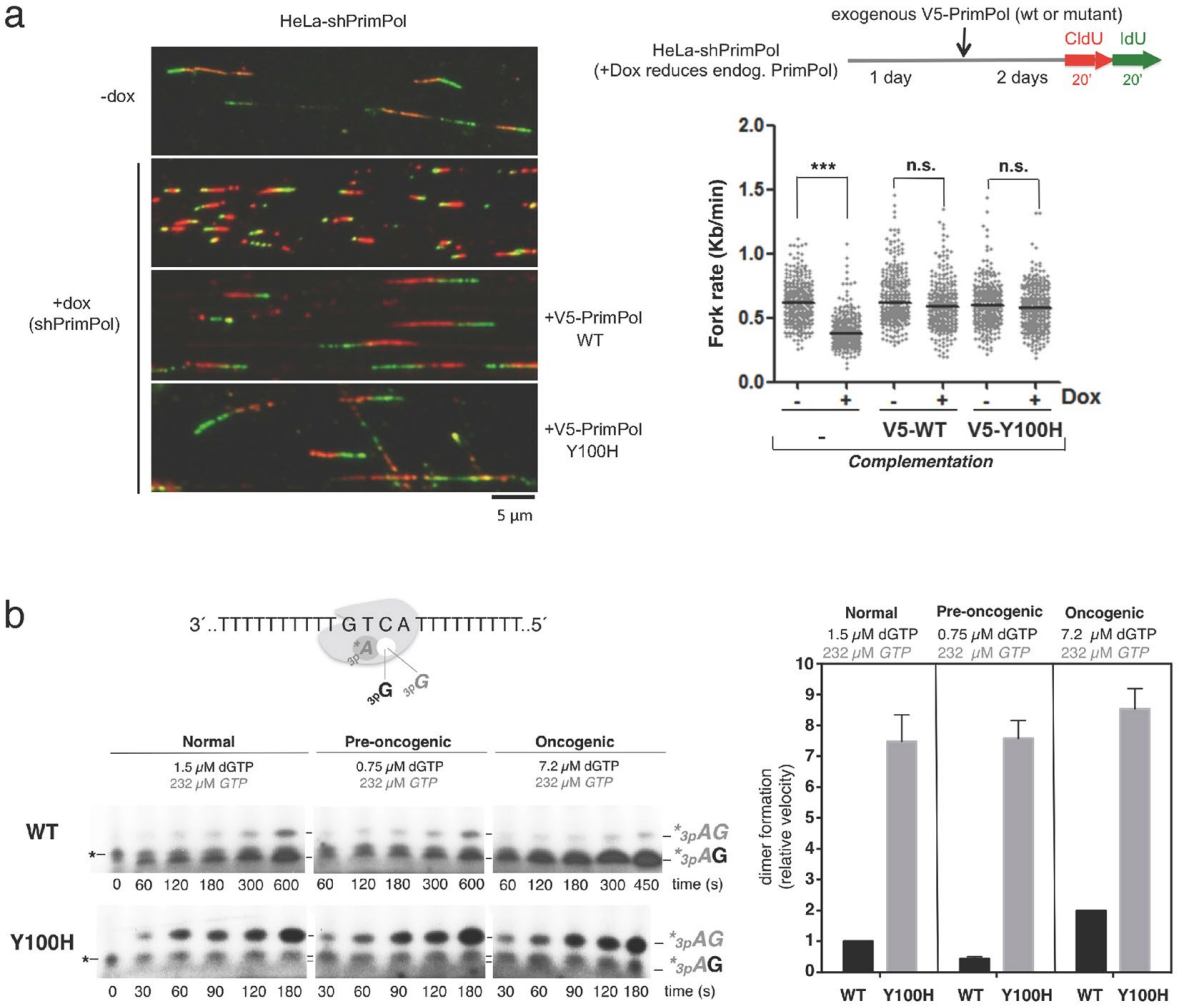
Y100H variant is competent for re-priming *in vivo*. (**a**) Workflow of the experimental design to measure replication fork rate by DNA fiber analysis after downregulation of endogenous PrimPol and expression of exogenous wild-type (WT) PrimPol or Y100H mutant. Fork rate values were calculated from the green length of red-green tracks. N > 300 values in each condition; n.s.: non-significant; ***p < 0.001 (Mann-Whitney test). Representative images of the different conditions are shown. (**b**) Dimer formation (primase assay) carried out using a 29-mer DNA template GTCA by either WT PrimPol or Y100H, in the presence of [γ-^32^P]*ATP* (16 nM) as the 5′nucleotide, and different physiological concentrations of either dGTP and *GTP*, as the incoming 3′nucleotide (normal cell concentration: 1.5 µM dGTP + 232 µM *GTP*, pre-oncogenic cell: 0.75 µM dGTP + 232 µM *GTP* or oncogenic cell: 7.2 µM dGTP + 232 µM *GTP*). The histogram shows the relative velocity of total dimer formation (*A*dG and *AG*) in the primase assays shown at the left. Full length gels corresponding to b are shown in Supplemental Fig. 7. The autoradiographs shown in this figure are representative of at least 3 independent experiments.

PrimPol assists DNA replication by re-priming ahead of stalled forks to reinitiate DNA synthesis^24–26,43^, and this is arguably its main physiological role. The importance of this function is revealed by a drop in the rate of fork progression, as determined by DNA fiber analysis after doxycycline-mediated induction of PrimPol shRNA^24^; see also Fig. 4a). Normal fork rate can be re-established by ectopic expression of WT PrimPol (V5-WT in Fig. 4a), but not by a primase-deficient mutant^24^. Interestingly, ectopic expression of the Y100H mutant (V5-Y100H in Fig. 4a) also recovered normal fork rate, suggesting that a change in sugar selectivity affecting primer synthesis does not impede its normal function *in vivo*. Expression levels of WT and Y100H PrimPol versions were comparable in these experiments (see immunoblots in Supplemental Fig. 4a).

Inside cells, the concentration of nucleotides is highly asymmetrical, with the concentration of *NTPs* much higher than that of dNTPs^10^. Given that the Y100H variant lacks discrimination against *NTPs*, it would expected to be more proficient than WT PrimPol when making primers under physiological concentration of nucleotides. To test this idea, we estimated primase efficiency by quantifying the dimers formed on the 3′ (T)_n_GTCA(T)_n_ 5′ template when providing [γ-^32^P]*ATP* as the initiating nucleotide and different ratios of dGTP and GTP as competing 3′ nucleotides. Importantly, *3pA*dG and *3pAG* dimers formed in the same reaction could be resolved after electrophoresis due to the lower mobility of ribonucleotides *versus* deoxynucleotides (Fig. 4b). Given that the Y100H mutant was identified in a tumor sample, we considered three different deoxy/ribo ratios, according to published data^10,44^: normal (1.5 µM dGTP + 273 µM *GTP*); pre-oncogenic (0.75 µM dGTP + 273 µM *GTP*), in which dNTP pools are below normal concentrations^44^; and oncogenic (7.2 µM dGTP + 273 µM *GTP*), in which dNTP levels are increased over normal ratios^10^. Under these three conditions –in which *GTP* is 38- to 364-fold more abundant than dGTP– WT PrimPol mainly generated *3pA*dG dimers (96%) due to its sugar selectivity against GTP, and the velocity of total dimer formation (calculated and normalized as described in Methods) was proportional to the concentration of dGTP (black bars in the histogram of Fig. 4b). Strikingly, the Y100H tumoral variant showed a complete switch from dGTP to *GTP* preference even at the highest (oncogenic) dGTP:*GTP* ratio, producing mainly *3pAG* dimers (80%). Moreover, the Y100H variant showed a 4.5- to 7-fold increase in the total dimer formation rate (grey bars in the histogram of Fig. 4b) which can be taken as a measurement of its increased primase proficiency.

### PrimPol Y100H enhances cellular resistance to dNTP pool depletion by reducing DSBs

**Figure 5:**
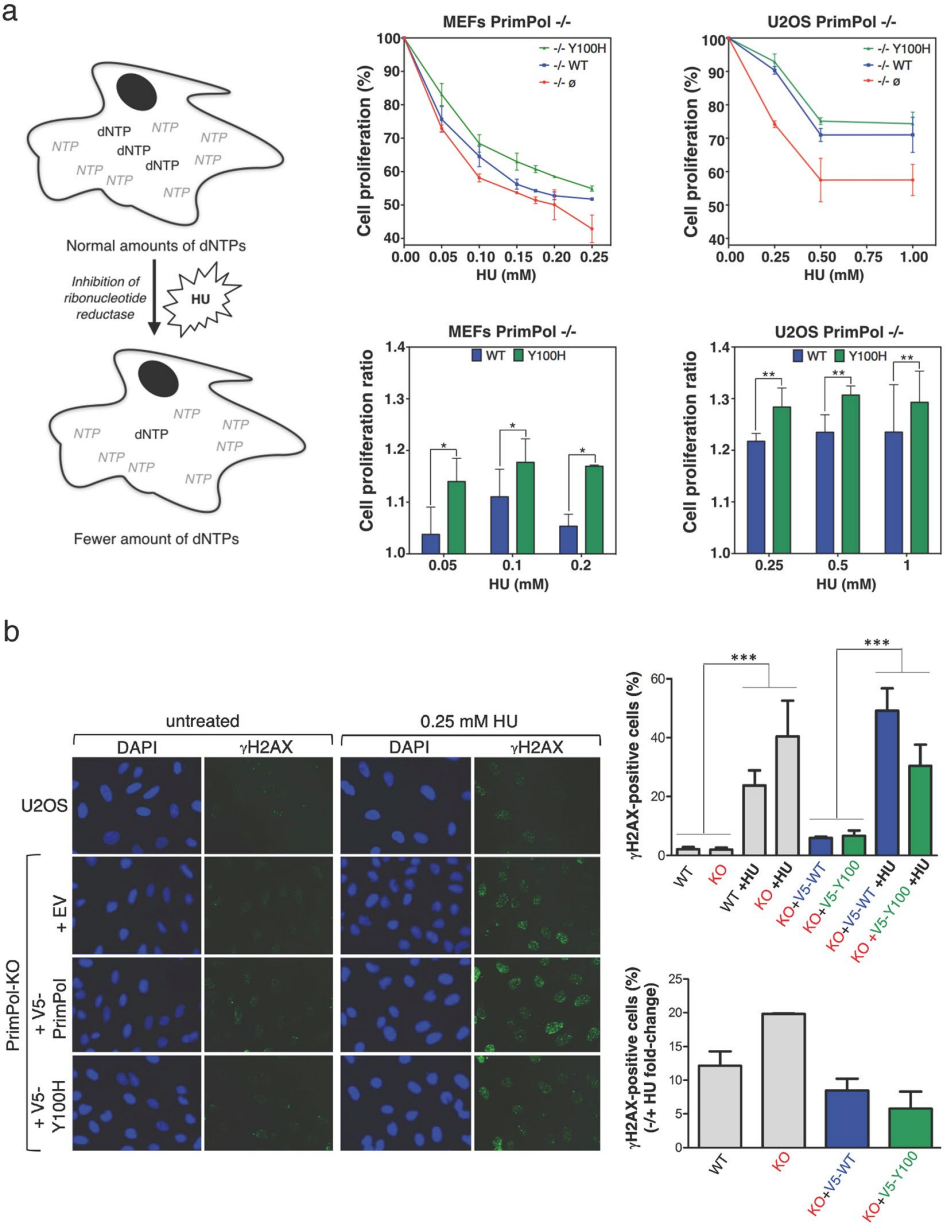
PrimPol Y100H enhances cellular resistance to dNTP pool depletion by reducing DSBs. (**a**) Left panels: schematic representation of the effect of hydroxyurea (HU) in altering the dNTPs/*NTPs* ratio. Central/upper panel: relative cell proliferation curves of PrimPol−/− MEFs transfected with empty vector (red), WT PrimPol (blue) or Y100H mutant (green), in the presence of increasing concentrations of HU (0.05, 0.10, 0.15, 0.20 and 0.25 mM); histogram in the central/lower panel shows the ratio of cell proliferation relative to WT PrimPol−/− cells at three HU concentrations (0.05, 0.1 and 0.2 mM). Right upper panel: relative cell proliferation curves of PrimPol−/− U2OS cells transfected as described above, in the presence of increasing concentrations of HU (0.25, 0.5 and 1 mM); the histogram in the right lower panel shows the cell proliferation ratio relative to WT PrimPol−/− cells at the same HU concentrations. t test **p < 0.01. (**b**) Left: representative confocal microscopy images of DAPI and γH2AX stainings in U2OS cells or PrimPol KO cells transfected with empty vector, WT PrimPol or Y100H mutant. When indicated, cells were incubated with 0.25 mM HU. Right: top histogram indicates the average median value of γH2AX intensity in each condition, derived from three biological replicates (>100 cells scored per condition in each replicate). Statistical significance was assessed with ANOVA and Bonferroni post-test. All pair-wise differences between lanes 1–2 and 3–4, or between 5–6 and 7–8, were significative. Bottom histogram depicts the fold-change difference in the intensity of γH2AX staining in the presence or absence of HU in each case.

The results shown above suggest that the Y100H variant is more proficient in cells than its WT counterpart, especially when dNTP levels are decreased, as it occurs in pre-oncogenic stages or following HU treatment (Fig. 5a, left panel). In agreement with previous reports in DT40 and HeLa cells^39,42^, MEFs lacking PrimPol were more sensitive to chronic HU treatment (Supplemental Fig. 4a). As expected, Y100H ectopic expression in PrimPol-deficient MEFs significantly enhanced (up to 3-fold) resistance to chronic HU treatment when compared with the expression of similar amounts (Supplemental Fig. 4c) of WT PrimPol (Fig. 5a, central panels). To confirm these results, we used PrimPol-ablated U2OS osteosarcoma cells, which were also more sensitive to HU chronic treatment (Supplemental Fig. 4b). Again, ectopic expression of the Y100H variant in these cells enhanced their resistance to chronic HU treatment to slightly higher levels than WT PrimPol expressed at similar amounts (Fig. 5a, right panels; Supplemental Fig. 4d). To determine if this effect is specific to HU, we tested if the Y100H variant could promote a resistant phenotype to aphidicolin, an inhibitor of replicative polymerases from family B, as Polα, Polδ and Polε, and therefore causative of DNA replication stress. Firstly, we showed that aphidicolin decreased cell proliferation to a larger extent in PrimPol-KO than in PrimPol-WT MEFs (Supplemental Fig. 4e). However, unlike the experiment with HU, ectopic expression of PrimPol-Y100H rescued wild-type levels of sensitivity to aphidicolin (Supplemental Fig. 4f). Thus, the observed specificity for HU directly correlates the gain in using ribonucleotide substrates by Y100H with the selective deprivation of dNTPs induced by HU.

Replication forks stalled by HU frequently lead to double-strand breaks (DSBs) particularly in rapidly dividing cells. Thus, we wondered if Y100H-expressing cells accumulate less DSBs, which could explain their higher resistance to HU. As expected, PrimPol-KO cells accumulated larger amounts of γ-H2AX foci (a marker of DSBs) than PrimPol-WT cells upon exposure to HU (Fig. 5b). Interestingly, ectopic expression of PrimPol-Y100H in HU-treated U2OS/PrimPol^−/−^ cells significantly reduced γ-H2AX foci compared to cells expressing PrimPol-WT. This experiment suggests that in cells challenged by HU, and therefore deprived of dNTPs, the Y100H variant can use NTPs to fulfill its re-priming function, thus avoiding persisting RS and subsequent DSBs.

In conclusion, the specific advantage of the Y100H variant to generate primers, especially when dNTPs are limiting, could have been positively selected to alleviate the intense RS that is characteristic of many tumoral cells.

## Discussion

Most DNA polymerases efficiently discriminate against *NTP* incorporation by virtue of an amino acid residue that acts as a steric gate, which, because of its bulky side chain, can sterically clash with the 2′-OH group of the incoming *NTP*, thereby hindering its binding and/or incorporation^8,45^. PrimPols from the different kingdoms of life are exceptional primases as they favor dNTPs over *NTPs*, as shown in Archaea^31,32^, Bacteria^46,47^ and Eukarya^23^. In this work we have identified human PrimPol Tyr^100^ as a mediator of sugar discrimination; however, our data suggest that it may not operate as a conventional steric gate as PrimPol does not discriminate against *NTP* incorporation as efficiently as other DNA polymerases such as replicases. Indeed, our results indicate that the Tyr^100^ side chain is not only involved in steric clashing with the incoming *NTP’s* 2′-OH group, but it is also important for the architecture of the PrimPol active site and consequently for PrimPol activity. Accordingly, the mutation of Tyr^100^ to histidine enhanced *NTP* incorporation, but also decreased dNTP incorporation in most of our experiments, reflecting its relevance for the overall activity of PrimPol. Interestingly, the crystal structure of *Mt*PolDom, an AEP member with high affinity for *NTPs*, reveals a histidine residue located at an equivalent position to Tyr^100^ that interacts with the 2′-OH of the incoming *NTP* (Fig. 1), suggesting that it favours the incorporation of these substrates. Indeed, the Y100H mutation dramatically increased the binding affinity of human PrimPol for *NTPs*, enhancing their incorporation in all *in vitro* reactions tested, and impacting PrimPol primase and TLS activities. Accordingly, the position occupied by Tyr^100^ appears to define sugar discrimination among AEP enzymes: those harbouring a tyrosine –such as *Hs*PrimPol and *Pfu-*p41– likely favour dNTP incorporation, whereas members harbouring a histidine can proficiently incorporate *NTPs*.

The main role of PrimPol as a specific primase that mediates replication restart in eukaryotes poses the intriguing question of why, unlike conventional primases, does PrimPol discriminate against *NTP* incorporation? In other words, what is the advantage of making DNA primers over *RNA* primers? This could be possibly related to PrimPol efficient polymerase and TLS activities, a critical difference between PrimPols and other primases. It is worth noting that the *RNA* primers generated by the conventional eukaryotic primase must be converted by Polα into “DNA primers”, before they can be efficiently extended by Polδ and Polε. Conversely, PrimPol action is simplistic, as it provides a more ergonomic solution during unscheduled priming: the direct synthesis of a DNA primer, which is ready for elongation by a replicase. Another relevant question is whether the DNA primers made by PrimPol are partially removed or edited by the mismatch repair system; if this is not the case, PrimPol action could impose a certain level of mutagenesis, including bases substitutions and indels^39^ or the sporadic and limited insertion of ribonucleotides. It is tempting to speculate that lagging strand primases evolved from PrimPols by opening the steric gate, to validate *NTPs* as substrates. Thus, the more homogenous nature of the primers (all *RNA*) made by conventional primases could facilitate their elimination, minimizing mutagenesis, especially during lagging strand synthesis.

It is generally accepted that *NTP* incorporation and persistence in the DNA is a harmful situation for cells that is counteracted by the ribonucleotide excision repair (RER) pathway. This pathway operates in the nucleus, but not in mitochondria^48^, which explains the abundance of embedded *NMPs* in mitochondrial DNA. Several pathologies are related to the alteration of mechanisms that prevent *NTP* incorporation or remove inserted *NTPs*, due to: (1) an increase in the *NTP*/dNTP ratio inside the nucleus and/or mitochondria, as occurs by aberrant activation of the retinoblastoma (Rb) E2F pathway (Rb-E2F), by either viral (HPV-16 E6/E7) or cellular (*cyclin E*) oncogenes^44^, or in Mpv17-related disorders that provoke a low dGTP concentration in liver mitochondria^49^; (2) mutation of specific components of the RER pathway, thus allowing persistence of embedded *NMPs* into DNA causing a severe phenotype as occurs in the RNase H2-deficient mouse^12,50^. In addition, it is also possible that specific mutations affecting the steric gates of DNA polymerases could have a pathological effect, mediated by an increase in embedded *NMPs* into DNA. We show here that human PrimPol can be converted into a “conventional” primase just by a single mutation (Y100H) at the sugar selector residue Tyr^100^. The RNA primers made by the Y100H variant appear to be functional to re-start stalled replication forks, and are synthesized with a higher efficiency specially in conditions of dNTP deprivation. These observations pose some intriguing questions: are these Y100H/PrimPol-made RNA primers eliminated as normal RNA primers of the lagging strand, or could they be targeted by the RER pathway? If not, would they contribute to genome instability? Interestingly, the Y100H mutation has been described in tumour cells and is compiled in the COSMIC database^30,38^. This could suggest that ablating the ability of PrimPol to discriminate against *NTPs* could promote survival during tumorigenesis. Accordingly, Y100H expression in MEFs or U2OS cells increased resistance against dNTP partial depletion with chronic sublethal treatment with HU, which mimics incipient tumorigenesis stages where dNTP concentration is low^44^. It is therefore tempting to speculate that the Y100H mutation could be selected early in tumorigenesis to deal with low dNTP levels, and to preclude DSBs caused by persistent replicative stress. Finally, given the dramatic effect on the dNTP/*NTP* discrimination factor of PrimPol variant Y100H, a relevant question would be wether the frequency of embedded ribonucleotides is increased in cells carrying the Y100H mutation at pretumoral lesions. If so, this could have caused the genomic instability that led to the cancerous state.

Finally, expression of the Y100H mutant in an RER-deficient background could be a valuable experimental approach to further explore the processes underlying replication re-start in mammals, and the role of PrimPol during mitochondrial DNA replication – the mechanistic details of which are still under debate^48,51^.

## Materials and Methods

### Primary sequence alignments, structure visualization and modelling of AEP primases

Multiple sequence alignments were performed using COBALT (constraint-based multiple alignment tool)^52,53^ from the National Center for Biotechnology Information (NCBI). Three-dimensional images were created with the PyMol Molecular Graphics System (version 1.2r3pre, Schrödinger, LLC) using *Hs*PrimPol PDB ID: 5L2X^34^, *Pfu*-p41 PDB ID: 1G71^33^, and *Mt*PolDom PDB ID: 3PKY^36^. A ternary complex of *Pfu*-p41 with DNA template and primer strands and incoming nucleotide was modeled by fitting *Hs*PrimPol PDB ID: 5L2X to *Pfu-*p41 PDB ID: 1G71 (using the three invariant catalytic aspartates (motifs A and C) and the invariant histidine (motif B) as common coordinates). A ternary complex of *Mt*PolDom was modelled by combining the protein structure and the incoming nucleotide from PDB ID: 3PKY, and DNA template/primer from PDB ID: 4MKY^37^. Both PDB structures of *Mt*PolDom were fitted by aligning the three invariant catalytic aspartates (motifs A and C) and the invariant histidine (motif B).

### Oligonucleotides, nucleotides and antibodies

DNA and RNA oligonucleotides were synthesized by Sigma Aldrich (St Louis, MO, USA). Unlabelled ultrapure dNTPs were supplied by GE (Fairfield, CT, USA). Radiolabeled nucleotides [γ-^32^P]*ATP*, [α-^32^P]dATP and [α-^32^P]dGTP (3000 Ci/mmol) were obtained from Perkin Elmer (Waltham, MA, USA). T4 polynucleotide kinase used for 5′ labeling of oligonucleotides was supplied by New England Biolabs (Ipswich, MA, USA). Anti-human PrimPol antibody (1:1000 dilution) was generated by ThermoFisher (Waltham, MA, USA). Secondary antibody ECL^TM^ Anti-Rabbit IgG was detected by Luminata^TM^ Forte Western HRP Substrate in MEFS (GE Healthcare). PrimPol in U2OS cells was detected by LI-COR secondary antibodies IRDye 800CW and IRDye 680RD using Odyssey equipment.

### Mutagenesis and enzyme purification

The coding Y100H point mutation in the WT PrimPol gene was introduced by PCR using the QuickChange site-directed mutagenesis kit (Stratagene, San Diego, CA, USA), using the vector pET16b:CCDC111^23^ as template and the following primers: 5′CAGAAAAAATCTCTTACACTGCCATGAAGTTATTCCTGAAAATGC-3′ and 5′-GCATTTTCAGGAATAACTTCATGGCAGTGTAAGAGATTTTTTCTG-3′. Overexpression and purification of both WT PrimPol and Y100H variant was carried out as previously described^23^.

### Polymerase assay on specific template/primer molecules

Oligonucleotides used as primers were labelled with PNK and [γ-^32^P]*ATP* as indicated by the manufacturer, and hybridized with the corresponding template in a 1:2 stoichiometry, in 50 mM Tris pH 7.5 and 300 mM NaCl. The following template/primer structures were used: (1) 28-mer DNA template (5′-AGAAGTGTATCXCGTACTCACTGTGATC-3′, where X is dA, dC, dG or dT) / [γ-^32^P]-labelled 15-mer DNA primer (5′-GATCACAGTGAGTAC-3′) or RNA primer (5′-*GAUCACAGUGAGUAC*-3′); (2) 34-mer DNA template (5′-GTACCCGGGGATCCGTACXGCGCATCAGCTGCAG-3′)/[γ-^32^P]-labelled 15-mer DNA primer (5′-CTGCAGCTGATGCGC-3′ where X is dG, dT or 8oxoG); (3) 45-mer DNA template with a CPD thymine dimer at T = T (5′-AGGATAGTGACAGTAGTGTATGT = TATAGTCAGTATGATCGTACGG-3′)/[γ-^32^P]-labelled 15-mer DNA primer (5′-CCGTACGATCATACTGACTAT-3′).

The reactions (20 μL) were carried out in buffer R (50 mM Tris-HCl [pH 7.5], 50 mM NaCl, 1 mM MnCl_2_, 1 mM DTT, 2.5% glycerol and 0.1 mg/ml bovine serum albumin) with 2.5 nM template/primer DNA, increasing concentrations of dNTPs or *NTPs* and 200 nM of either WT PrimPol or Y100H mutant, unless indicated otherwise. After 20 min at 30 °C, reactions were stopped by the addition of formamide loading buffer (95% formamide, 10 mM EDTA, 0.1% bromophenol blue and 0.1% xylene-cyanol) and loaded in and 8 M urea-containing 20% polyacrylamide sequencing gel. Autoradiography was used to detect primer extension products.

### Steady-state kinetics assay

Kinetic parameters of + 1 nt primer extension reactions mediated by either WT PrimPol or mutant Y100H were analyzed as described previously^17,54^. The reaction mixture (20 µL) contained, in buffer R: 0.2 M of the 28-mer template/primer structure described above and 40 nM WT PrimPol or Y100H variant. Reactions were incubated at 30 °C during 7 min in the case of WT PrimPol with dNTPs and Y100H with dNTPs or *NTPs*. WT PrimPol was incubated with *NTPs* during 10 min at 30°C. Reactions were stopped and resolved as described above. Gel band intensities were analyzed using a BAS reader 1500 (Fujifilm). Autoradiographs were quantitated by densitometry using ImageJ software (NIH). The observed rate of nucleotide incorporation (extended primer) was plotted as a function of nucleotide concentration. Data were fit to the Michaelis–Menten equation using non-linear regression to determine the apparent K_m_ and k_cat_ parameters.

### Primase assays on specific oligonucleotide templates

The reaction mixture (20 µL) contained, in buffer R: [α-^32^P]dATP or [γ-^32^P]*ATP* (16 nM) as 5′ site nucleotide, increasing concentrations of dGTP or *GTP* (or both) as 3′site nucleotide, in the presence of WT PrimPol or Y100H variant (400 nM). The oligonucleotides used as templates (1 µM) were: 29-mer 5′ -T_15_ACTGT_10_-3′ (abbreviated as GTCA) and 60-mer 5′-T_20_ACGACAGACTGT_29_ -3′. After 20 min at 30 °C, reactions were stopped as described above. When indicated, different reaction times were used to obtain velocities. After electrophoresis, *de novo* synthesized dimers were detected by autoradiography and quantitated by densitometry as described above.

### Electrophoretic mobility shift assays

Interaction of PrimPol and ssDNA template was carried out in buffer R (supplemented with 2.5% (w/v) PEG-4000), by mixing 1 nM of labelled GTCC oligonucleotide (5′ -T_36_CCTGT_20_-3′) and increasing concentrations of WT PrimPol or Y100H variant. Reactions were incubated for 10 min at 30 °C and then stopped by adding loading buffer (50% glycerol, 1 mM EDTA, 0.1% xylene cyanol and 0.1% bromophenol blue). Immediately, reactions were loaded in a native 6% polyacrylamide gel, and run at 150 V at 4 °C for 120 min in Tris-glycine pH 8.3 buffer. After electrophoresis, the binary complex protein/DNA and free DNA were detected by autoradiography. EMSA for pre-ternary complex formation was carried in buffer R with 2.5% (w/v) PEG-4000, non-labelled oligo GTCC (500 nM), WT PrimPol or Y100H mutant (1 µM), and the labelled nucleotide [α-^32^P]dGTP or [α-^32^P]*GTP* (16 nM). Mixtures (20 µL) were incubated for 30 min at 30 °C. Then, loading buffer was added and enzyme:DNA:d/*NTP* pre-ternary complex was analyzed as described above.

### Single-molecule analysis of DNA replication in stretched DNA fibers

The stable cell line HeLa-shPrimPol has been described before^24^. DNA sequences encoding WT PrimPol and Y100H variant were cloned into Gateway expression vectors (Invitrogen) introducing an N-terminal V5 tag. Transient transfection was performed using Lipofectamine 2000 (ThermoFisher, Waltham, MA, USA). HeLa-shPrimPol cells growing exponentially in culture were pulse-labelled with 50 μM CldU (20 min) followed by 250 μM IdU (20 min). Labelled cells were harvested and resuspended in phosphate buffered saline at 0.25 × 10^6^ cells/mL. Stretched DNA fibers were prepared as described^55^ with minor modifications. A detailed protocol is available upon request. For immunodetection of labelled tracks, fibers were incubated with primary antibodies anti-CldU (rat monoclonal anti-BrdU, Abcam #AB6326) and anti-IdU (mouse monoclonal anti-BrdU, BD Biosciences #347580) for 1 h at RT and the corresponding Alexa Fluor-conjugated secondary antibodies (Invitrogen/Molecular Probes #A-11007 and A-21121) for 30 min, both at room temperature in a humidity chamber. DNA was stained with anti-ssDNA (Millipore, #MAB3034) to assess fiber integrity. Fiber images were obtained in a DM6000 B Leica microscope. Fork rate was estimated from > 300 red-green tracks per condition using conversion factor 1 μm = 2.59 kb^56^.

### Cell proliferation assays in the presence of hydroxyurea or aphidicolin

MEFs derived from PrimPol−/− mice and PrimPol−/− U2OS cells generated by CRISP-Cas9 editing were cultured in Dulbecco’s Modified Eagle Medium (Life Technologies, Carslbad, CA, USA) supplemented with 10% HyClone fetal bovine serum (ThermoFisher, Waltham, MA, USA) and 1% penicillin and streptomycin (Life Technologies, Carlsbad, CA, USA). Cells were seeded in p24 plates at 3 × 10^4^ cells per well and transfected with empty pcDNA3.1(-) or pcDNA:CCDC111 encoding WT PrimPol or the Y100H variant. TurboFect Transfection Reagent or Lipofectamine 2000 was used for MEFs and U2OS, respectively (ThermoFisher, Waltham, MA, USA). After 48 h, cells were treated with the indicated concentrations of HU (Sigma Aldrich, St Louis, MO, USA) during 24 h (MEFs) or 48 h (U2OS) or with APH (Sigma Aldrich, St Louis, MO, USA) during 24 h (MEFs). Cells were fixed with 10% formaldehyde for 30 min and relative cell proliferation was assessed by crystal violet staining.

### Quantification of DNA damage by γH2AX staining

WT U2OS or isogenic PrimPol KO cells, grown on DMEM-10% fetal bovine serum supplemented with penicillin/streptomycin, were transfected with 2 ug of pcDNA3.1-based plasmids expressing either V5-PrimPol or V5-PrimPol Y100H. 24 h after transfection, cells were seeded in Opera Greiner microclear plates (6000 cells/well). 0.25 mM HU was added to the medium for 48 h. Cells were pre-extracted for 5 min in ice with 0.5% Triton X-100 in CSK buffer (10 mM PIPES pH 7.0, 0.1 M NaCl, 0.3 M sacarose, 3 mM MgCl2, 0.5 mM PMSF) and fixed with 4% paraformaldehyde for 15 min. Immunofluorescence detection of γH2AX was preceded by a 30 min incubation in blocking solution (3% BSA in phosphate-buffered saline containing 0.01% Tween). γH2AX antibody (EMD Millipore 05–636; 1:200 in blocking solution) was added for 1 h at RT. Secondary antibody AF-488 anti-mouse IgG (1:300 in blocking solution) was added for 1 h at RT. Nuclei were counterstained with DAPI. Images were analyzed in an Opera high-content screening system (Perkin Elmer): γH2AX intensity was measured within the nuclei mask and quantified using Acapella software.

## Supplementary information

Supplemental Information
