# Supplementary material for: A cancer-associated point mutation disables the steric gate of human PrimPol
Source: Sci Rep. 2019 Feb 4;9:1121. doi: 10.1038/s41598-018-37439-0 (PMC6362072; doi:10.1038/s41598-018-37439-0)
Supplement: Supplementary file 1 — Supplemental Information [file 41598_2018_37439_MOESM1_ESM.pdf]

A cancer-associated point mutation disables the steric gate of human PrimPol

Supplemental information

Alberto Díaz-Talavera[1], Patricia A. Calvo[1], Daniel González-Acosta[2], Marcos Díaz[2], Guillermo Sastre-Moreno[1], Luis Blanco-Franco[1], Susana Guerra[1], Maria I. Martínez-Jiménez[1], Juan Méndez[2], and Luis Blanco[1]*

[1] Centro de Biología Molecular "Severo Ochoa" (CSIC-UAM) c/Nicolás Cabrera 1, Cantoblanco, 28049, Madrid, Spain.

[2] Centro Nacional de Investigaciones Oncológicas (CNIO), c/Melchor Fernández Almagro 3, 28029, Madrid, Spain.

* To whom correspondence should be addressed. Tel: +34 911964685; Fax: +34 911964401; Email: lblanco@cbm.uam.es

Supplemental Figure 1

***Hs*PrimPol, *Pfu*p41 and *Mt*PolDom orthologs.** Primary sequence alignment of the region encompassing the putative sugar selector residue and motif A in AEP-like members grouped as orthologs of human PrimPol (**a**), *Pfu*-p41 (**b**), and *Mt*PolDom (**c**), performed using the Basic Local Alignment Search Tool (BLAST) server from the National Library of Medicine. Catalytic residues involved in metal binding are indicated with red dots. Single residues acting as a sugar selector favouring *NTPs* or dNTPs are indicated with violet or pink dots, respectively. Secondary structure elements derived from 3D-structure analysis (PDB) are depicted above the alignments.

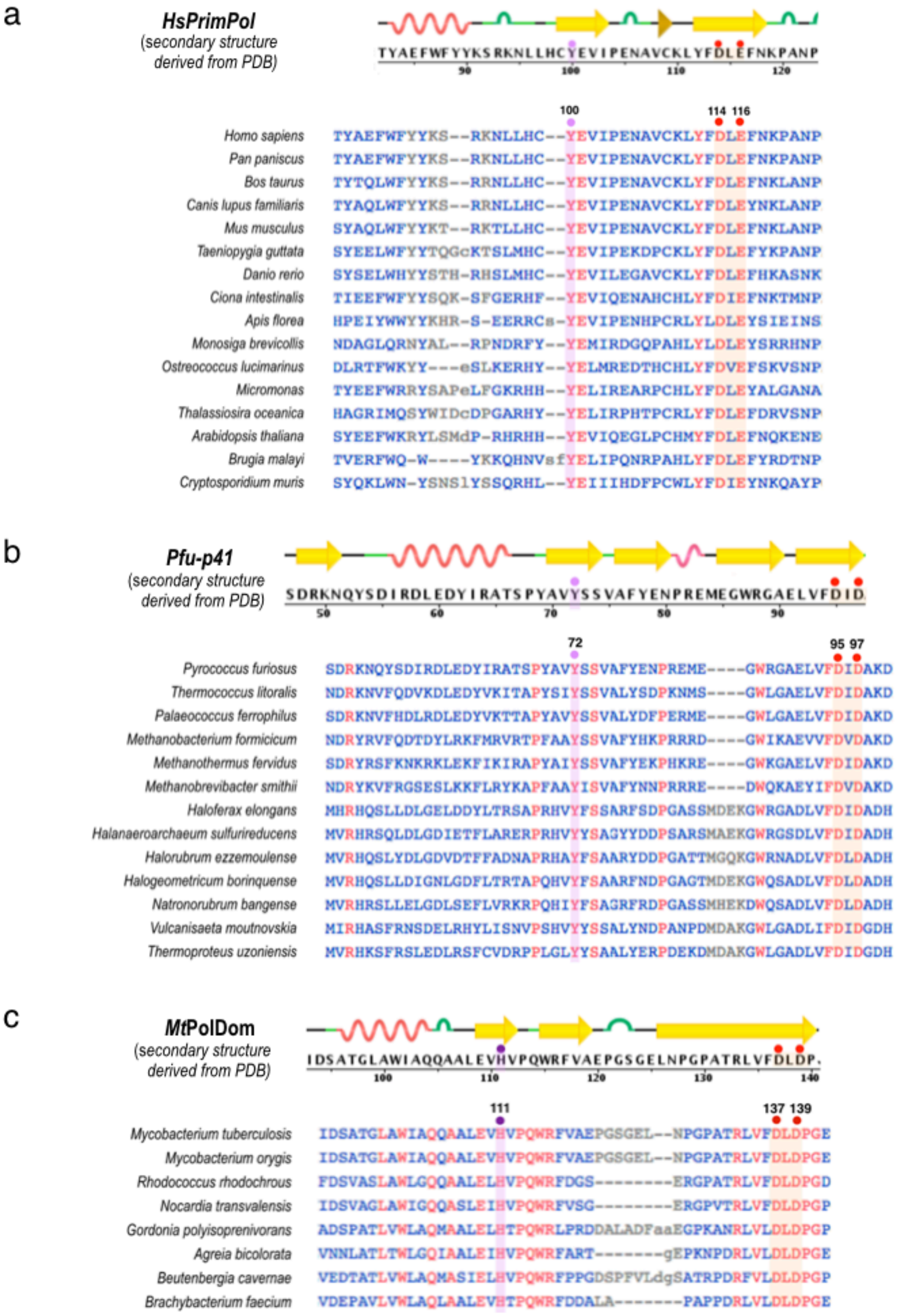

Figure S1 Díaz-Talavera et al.

Supplemental Figure 2

Misinsertion by Y100H, using physiological concentrations of ribonucleotides. At the top, a scheme of the 4 template/primer sequences used, differing in the first template base (X, stands either for dT, dC, dG or dA). Matched versus mismatched ribonucleotide insertion driven by the four template bases was comparatively analyzed by providing each of the 4 individual ribonucleotides. Ribonucleotide concentration used corresponded to the reported physiological amount of each nucleotide, as reviewed in: *Traut T.W. (1994) Physiological concentrations of purines and pyrimidines. Mol. Cell. Biochem. 140, 1-22*. The reaction was carried out in buffer R and 2.5 nM of each template/primer with WT PrimPol or Y100H (200 nM) in the presence of the indicated *NTPs*. Note that physiological ATP concentration (2,5 mM) inhibits PrimPol polymerase activity *in vitro,* therefore a 10- fold lower concentration was also included in the assay. The reactions were incubated at 30 °C during 20 min, and then analysed as previously described. Vertical dotted-lines were drawn above the autoradiography to separate the set of primer extension products obtained with each template/primer. Under these experimental conditions, WT PrimPol only produced the insertion of the complementary ribonucletide, opposite the templating base. Conversely, the Y100H variant produced a much more efficient incorporation of ribonucleotides, either complementary or not complementary to the first available templating base. The *in vivo* impact of these Y100H-mediated misincorporation events, if not repaired, remains to be determined.

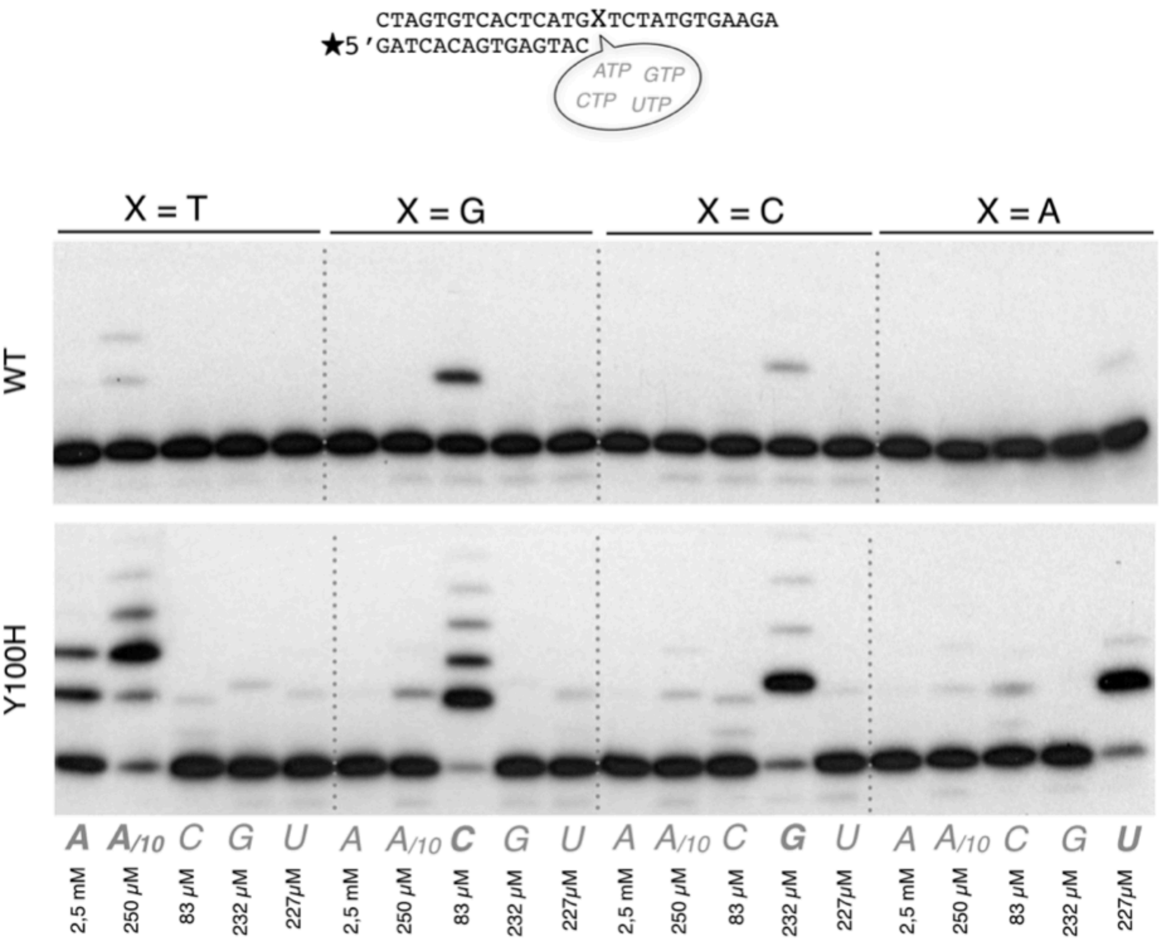

Figure S2 Díaz-Talavera et al.

Supplemental Figure 3

Y100H promotes primer realignment-mediated lesion skipping triggered by both dNTPs and *NTPs*. (**a**) A scheme of the labelled template/primer structures used, where X is T, abasic site (AP) or 6,4 photoproduct (6-4PP), is shown at the top. Primer elongation was evaluated in buffer R and 2.5 nM of each template/primer with WT PrimPol or Y100H (200 nM) in the presence of dNTPs or *NTPs* (1, 10, 50 and 100 μM). The reactions were incubated at 30 ºC during 30 min, then analyzed as previously described. The schemes at the bottom describe the observed reactions: conventional template-dependent primer elongation or different options of lesion skipping triggered by primer realignment. (**b**) Identification of the first and second nucleotide incorporated in each situation. The reaction was carried as described in methods, but using the indicated dNTPs or *NTPs* at 10 μM. Full length gels corresponding to part b are shown in Supplemental Fig. 8. The autoradiographs shown in this figure are representative of at least 3 independent experiments.

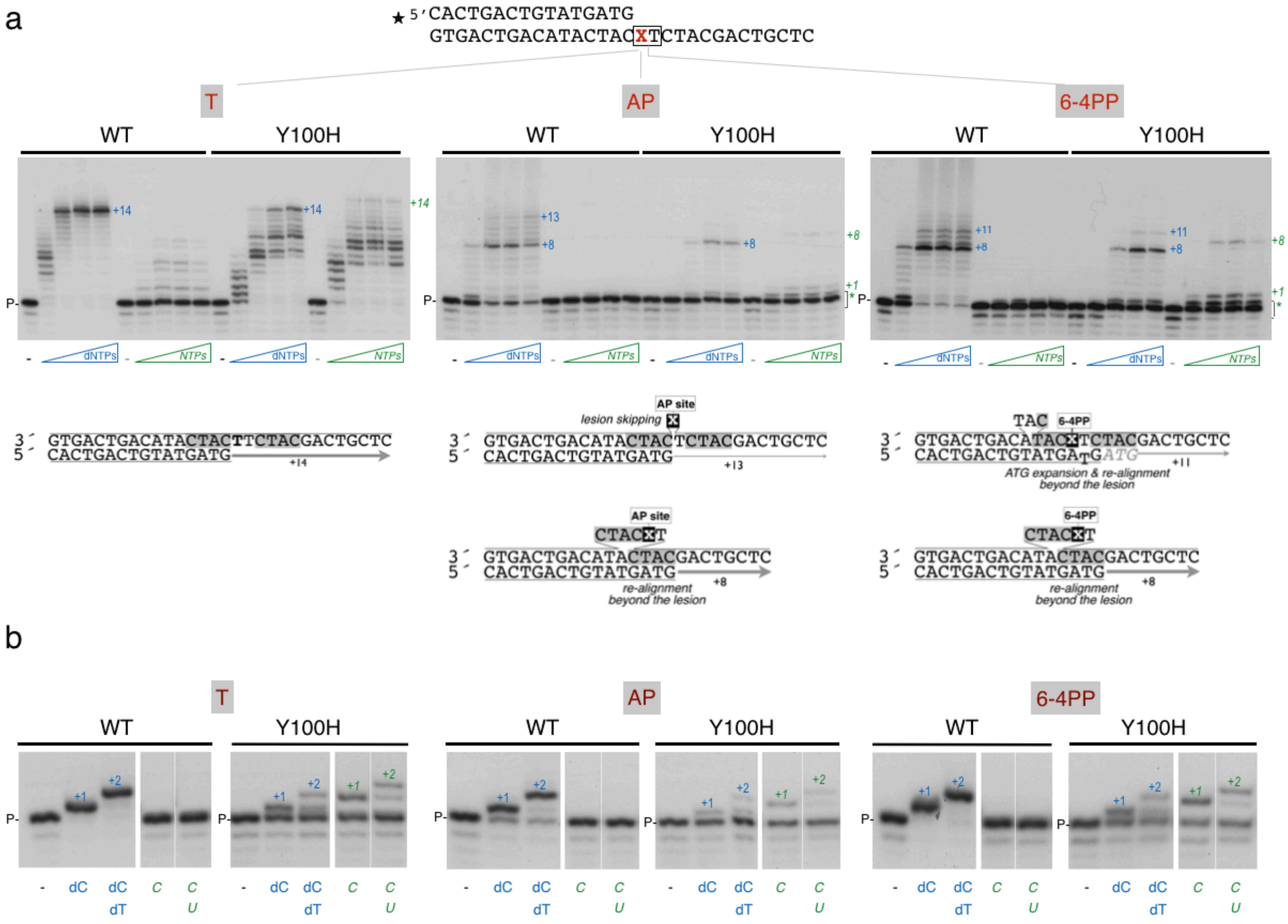

Figure S3 Diaz-Talavera et al.

Supplemental Figure 4

Effect of Y100H expression on cell proliferation in the presence of hydroxyurea. (**a**) Relative cell proliferation of PrimPol$^{+/+}$ (WT) or PrimPol$^{-/-}$ (KO) MEFs treated with the indicated concentrations of hydroxyurea (HU; 0.02, 0.03, 0.05, 0.1, 0.2, and 0.4 mM) during 24 h. Data are representative of at least 3 independent experiments. (**b**) Relative cell proliferation of PrimPol$^{+/+}$ (WT) or PrimPol$^{-/-}$ (KO) U2OS cells treated with different concentrations of HU (0.25, 0.5 and 1 mM) during 72 h. Data are representative of at least 3 independent experiments. (**c**) Immunoblot showing expression levels of exogenous WT PrimPol and Y100H after transfection of PrimPol$^{-/-}$ MEFs; Ponceau-S staining was used as loading control. **(d)** Immunoblot showing expression levels of exogenous WT PrimPol and Y100H after transfection of PrimPol$^{-/-}$ U2OS cells; actin was used as loading control. Full length gels and blots corresponding to parts c and d are shown in Supplemental Fig. 9. **(e)** Relative cell proliferation of PrimPol+/+ (WT) or PrimPol-/- (KO) MEFs treated with the indicated concentrations of aphidicolin (APH; 0.2, 0.5, and 1.0 µM) during 24 h. Data are representative of at least 3 independent experiments. **(f)** Relative cell proliferation curves of PrimPol-/- MEFs transfected with empty vector (red), WT PrimPol (blue) or Y100H mutant (green), in the presence of increasing concentrations of APH (0.2, 0.5, and 1.0 µM). Histogram in the right panel shows the ratio of cell proliferation relative to WT PrimPol-/- cells at two APH concentrations (0.5, and 1.0 µM). t test **p<0.01.

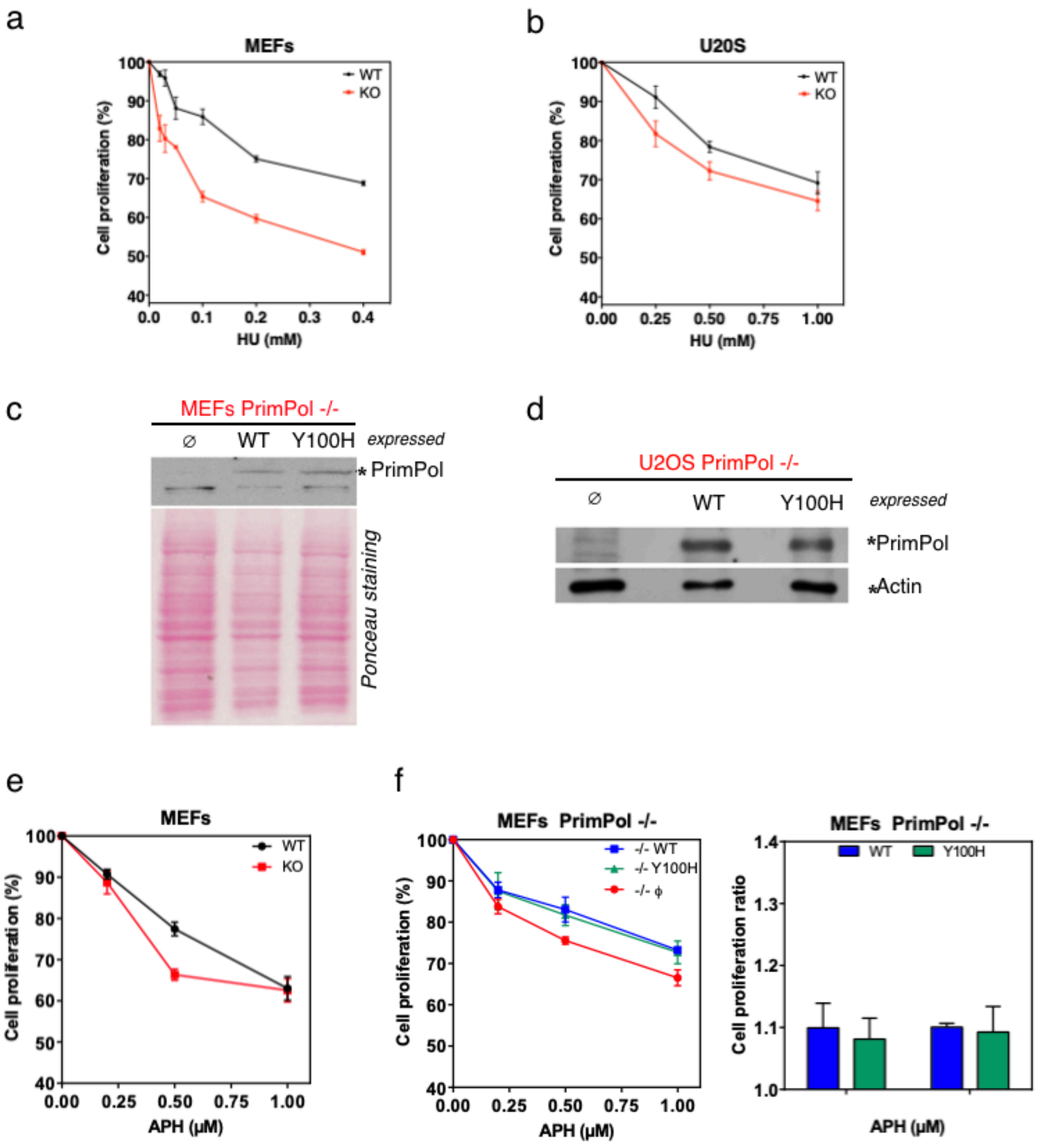

Figure S4 Díaz-Talavera et al.

Supplemental Figure 5

Full-length gels corresponding to the experiment shown in Fig. 2. The region of the gels cropped to compose the different parts of Fig. 2 are delimited with a red frame.

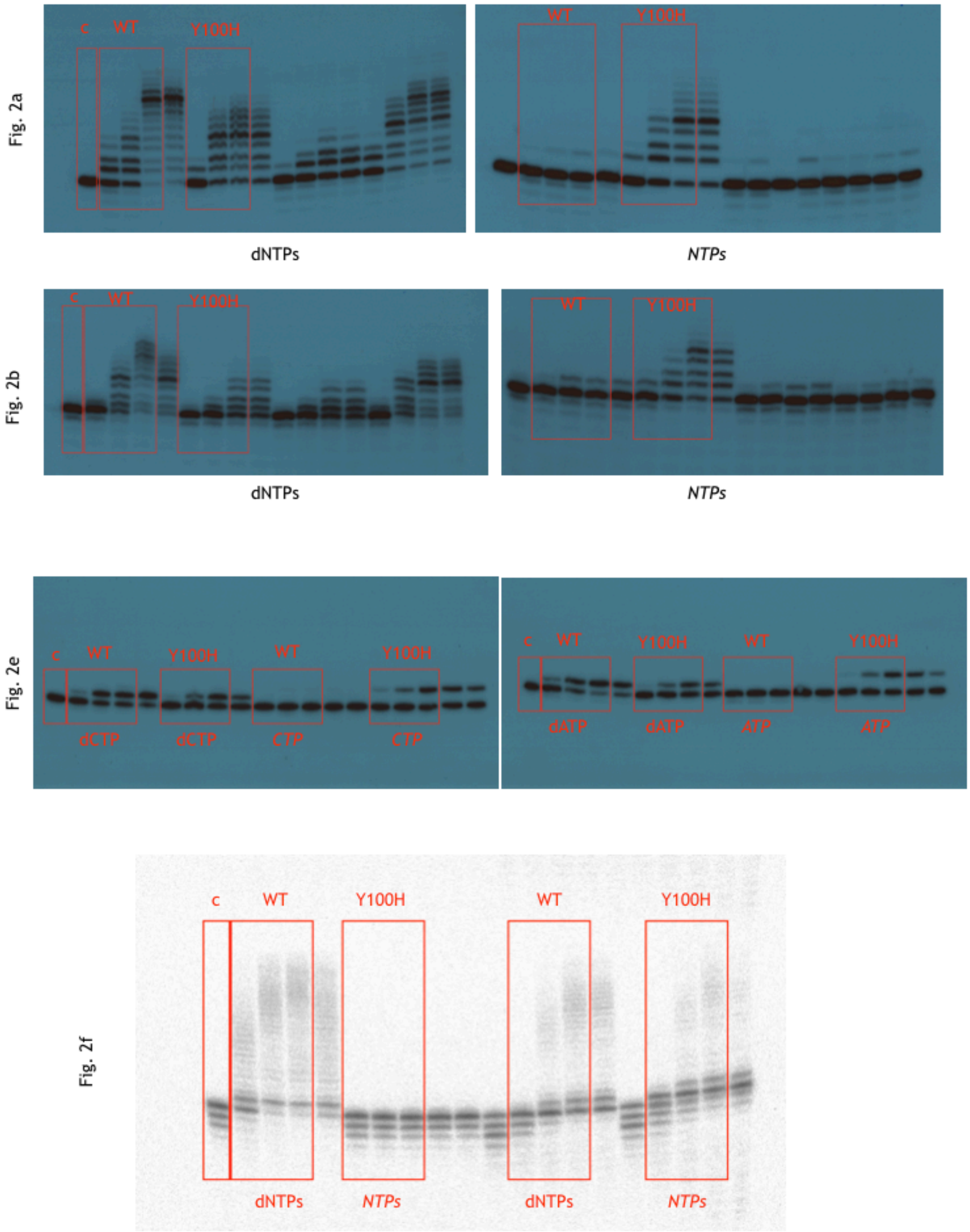

Figure S5 Díaz-Talavera et al.

Supplemental Figure 6

Full-length gels corresponding to the experiment shown in Fig. 3. The region of the gels cropped to compose the different parts of Fig. 3 are delimited with a red frame.

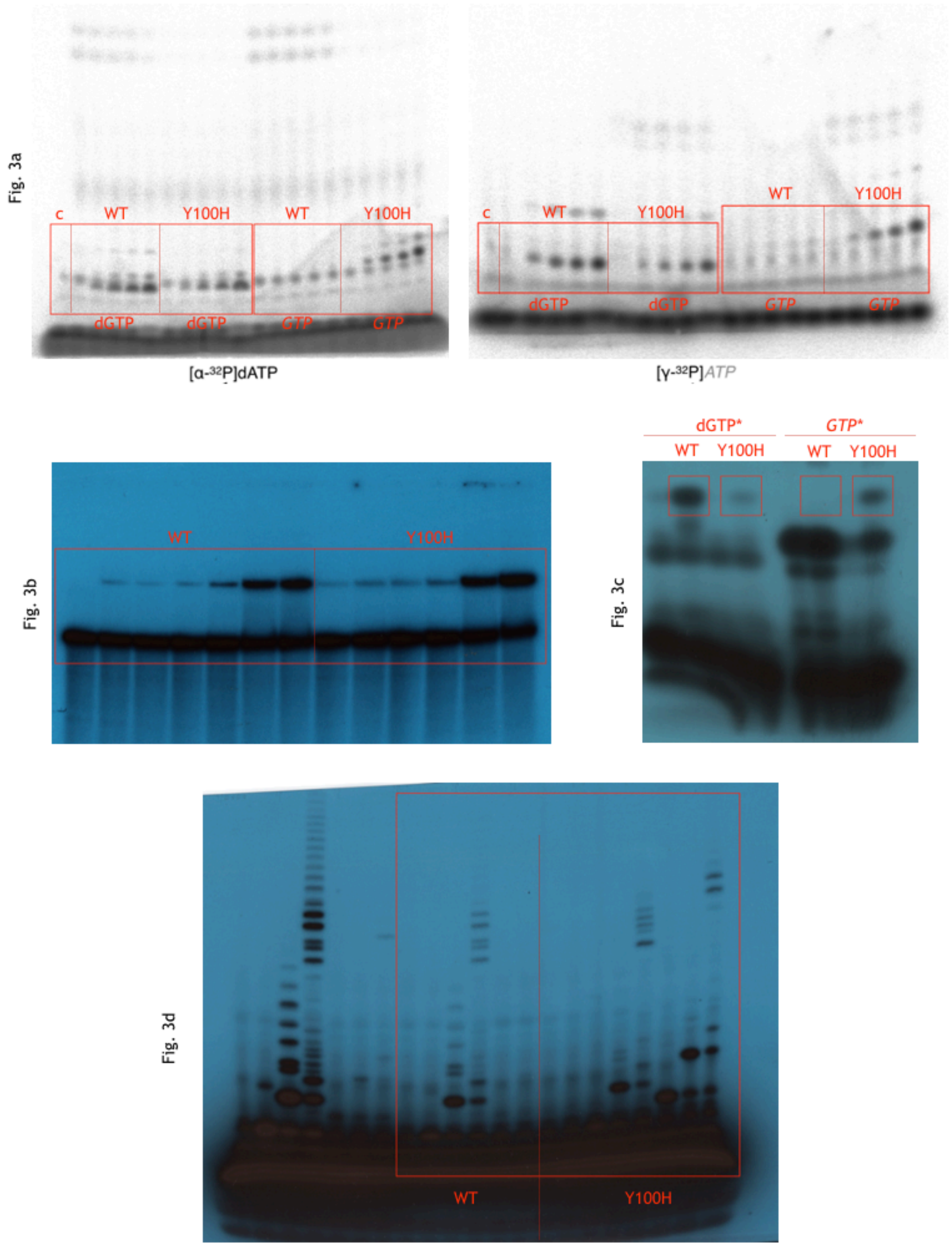

Figure S6 Díaz-Talavera et al.

Supplemental Figure 7

Full-length gels corresponding to the experiment shown in Fig. 4b. The region of the gels cropped to compose Fig. 4b are delimited with a red frame.

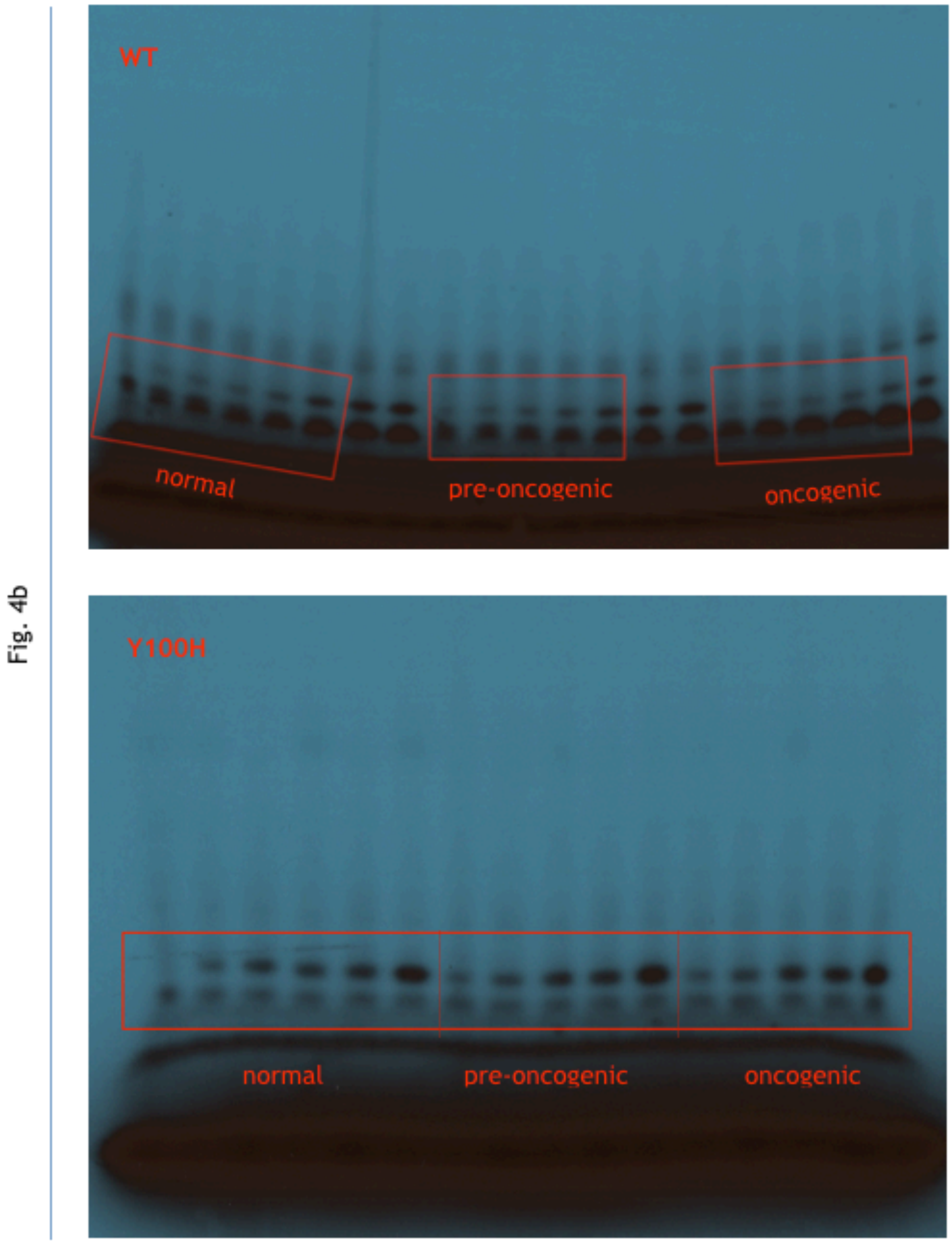

Figure S7 Díaz-Talavera et al.

Supplemental Figure 8

Full-length gels corresponding to the experiment shown in Supplemental Fig. 3b. The region of the gels cropped to compose Supplemental Fig. 3b are delimited with a red frame.

Fig. S3b

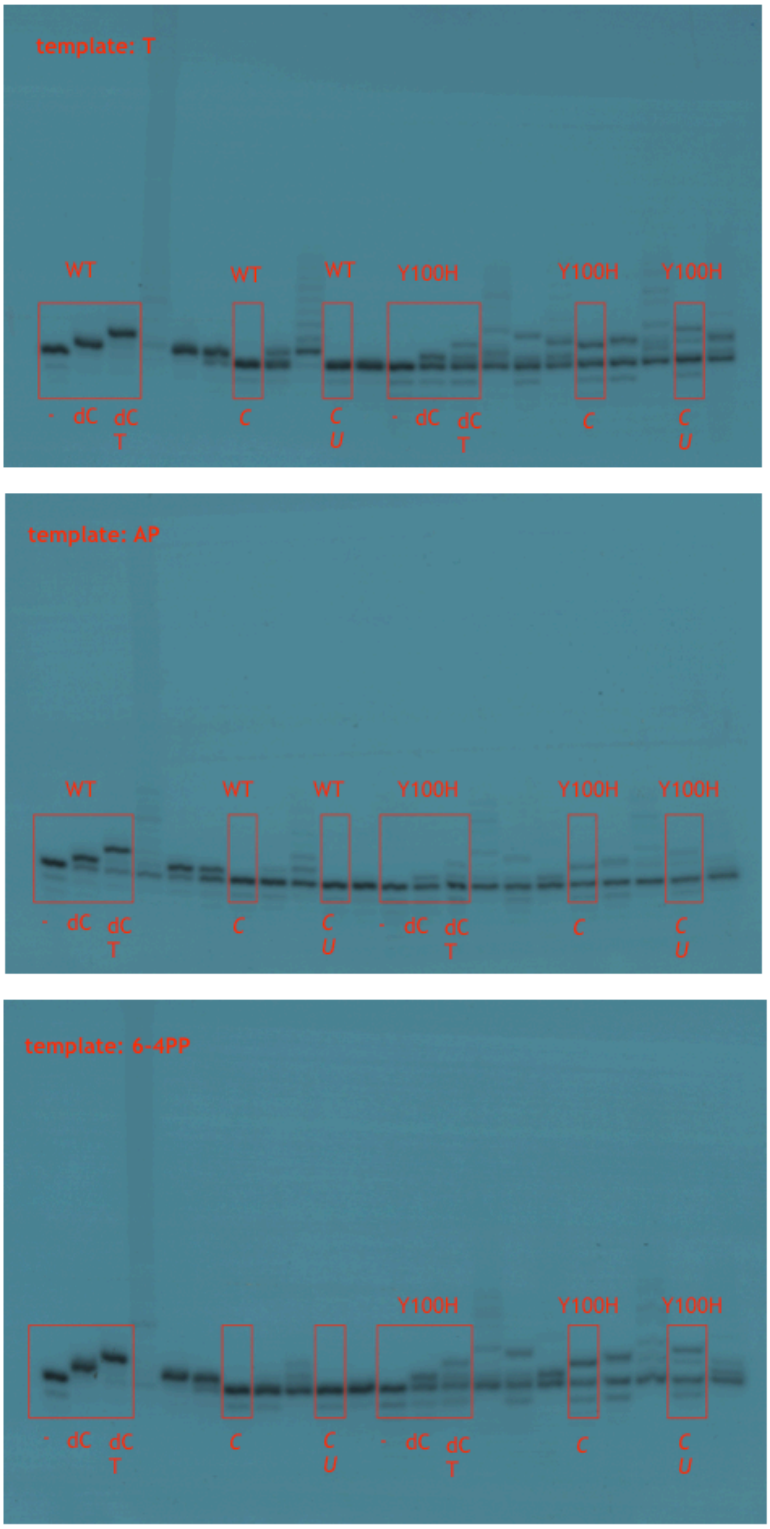

Figure S8 Díaz-Talavera et al.

Supplemental Figure 9

Full-length blots/gels corresponding to the experiment shown in Supplemental Fig. 4c & 4d. The regions cropped to compose Supplemental Fig. 4c & 4d are delimited with a red frame.

Fig. S4c

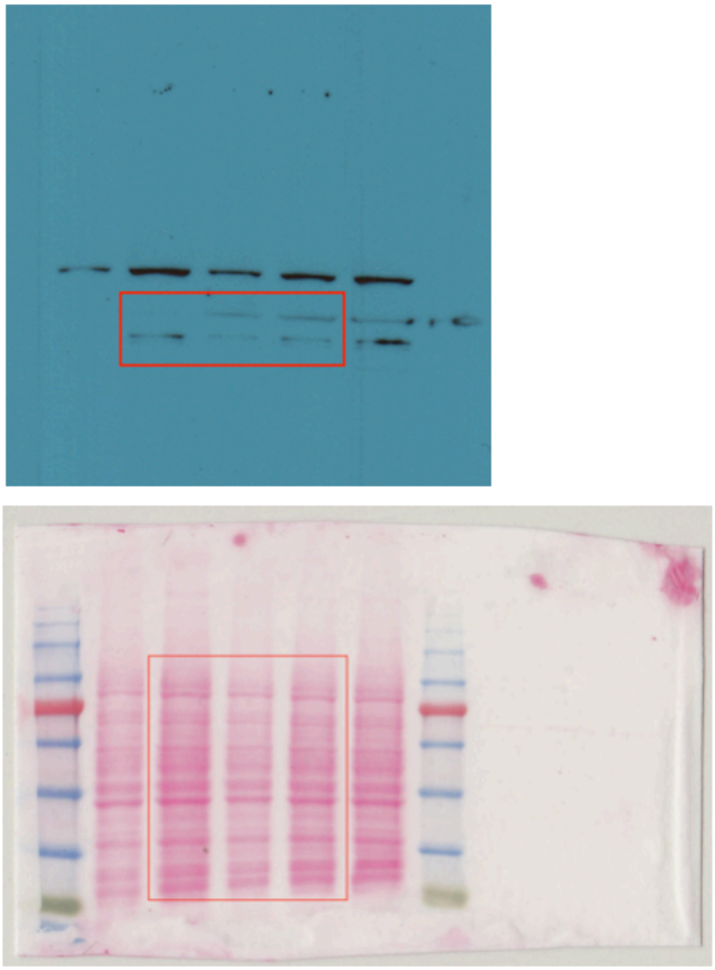

Fig. S4d

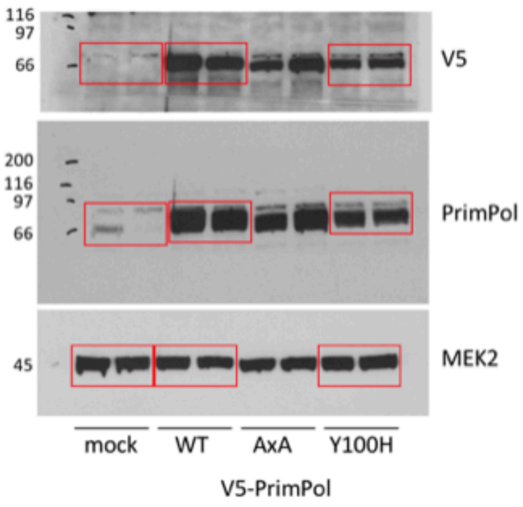

Figure S9 Díaz-Talavera et al.
